# IL-10-producing Th1 cells possess a distinct molecular signature in malaria

**DOI:** 10.1172/JCI153733

**Published:** 2023-01-03

**Authors:** Chelsea L. Edwards, Susanna S. Ng, Fabian de Labastida Rivera, Dillon Corvino, Jessica A. Engel, Marcela Montes de Oca, Luzia Bukali, Teija C.M. Frame, Patrick T. Bunn, Shashi Bhushan Chauhan, Siddharth Sankar Singh, Yulin Wang, Jinrui Na, Fiona H. Amante, Jessica R. Loughland, Megan S.F. Soon, Nicola Waddell, Pamela Mukhopadhay, Lambros T. Koufariotis, Rebecca L. Johnston, Jason S. Lee, Rachel Kuns, Ping Zhang, Michelle J. Boyle, Geoffrey R. Hill, James S. McCarthy, Rajiv Kumar, Christian R. Engwerda

**Affiliations:** 1 QIMR Berghofer Medical Research Institute, Brisbane, Australia.; 2 University of Queensland, School of Medicine, Brisbane, Australia.; 3 Griffith University, School of Natural Sciences, Nathan, Australia.; 4 Institute of Experimental Oncology, University of Bonn, Bonn, Germany.; 5 York Biomedical Research Institute, Hull York Medical School, University of York, York, United Kingdom.; 6 Department of Medicine, Institute of Medical Sciences, Banaras Hindu University, Varanasi, India.; 7 Clinical Research Division, Fred Hutchinson Cancer Research Centre, Seattle, Washington, USA.; 8 Victorian Infectious Diseases Services, Doherty Institute, University of Melbourne, Melbourne, Australia.; 9 Centre of Experimental Medicine and Surgery, Institute of Medical Sciences, Banaras Hindu University, Varanasi, India.

**Keywords:** Immunology, Infectious disease, Th1 response

## Abstract

Control of intracellular parasites responsible for malaria requires host IFN-γ^+^T-bet^+^CD4^+^ T cells (Th1 cells) with IL-10 produced by Th1 cells to mitigate the pathology induced by this inflammatory response. However, these IL-10–producing Th1 (induced type I regulatory [Tr1]) cells can also promote parasite persistence or impair immunity to reinfection or vaccination. Here, we identified molecular and phenotypic signatures that distinguished IL-10^–^Th1 cells from IL-10^+^Tr1 cells in *Plasmodium falciparum*–infected people who participated in controlled human malaria infection studies, as well as C57BL/6 mice with experimental malaria caused by *P. berghei* ANKA. We also identified a conserved Tr1 cell molecular signature shared between patients with malaria, dengue, and graft-versus-host disease. Genetic manipulation of primary human CD4^+^ T cells showed that the transcription factor cMAF played an important role in the induction of IL-10, while BLIMP-1 promoted the development of human CD4^+^ T cells expressing multiple coinhibitory receptors. We also describe heterogeneity of Tr1 cell coinhibitory receptor expression that has implications for targeting these molecules for clinical advantage during infection. Overall, this work provides insights into CD4^+^ T cell development during malaria that offer opportunities for creation of strategies to modulate CD4^+^ T cell functions and improve antiparasitic immunity.

## Graphical abstract

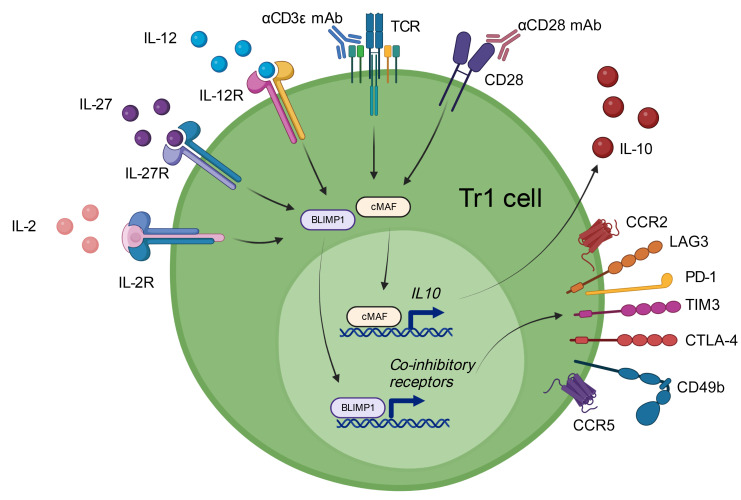

## Introduction

Under homeostatic conditions, inflammation is controlled by immunoregulatory networks that prevent disease (1). These networks comprise interactions among specialized immune cells, parenchymal cells, and tissue-specific microbiomes (2, 3). Following infection, immune cells are stimulated to produce inflammatory mediators required to control pathogen growth (4). However, once this growth is contained, these inflammatory responses contract to limit tissue damage and allow new immune responses to emerge as required (5). The magnitude and timing of inflammatory and regulatory immune responses generated during infection determines disease outcome, with the balance of these responses governing protection against reinfection (6, 7). Therefore, an improved understanding of the establishment of immunoregulatory pathways following infection and how they affect development of protective immunity is needed to facilitate disease prevention and control.

Specialized effector CD4^+^ T cell subsets are crucial for coordinating immune responses (8). Diseases caused by intracellular protozoan parasites such as malaria require dendritic cells and macrophages to present antigens that prime or expand IFN-γ^+^Tbet^+^CD4^+^ Th1 cells, which activate phagocytes to kill captured or resident pathogens (9). As inflammatory cytokines produced by Th1 cells can cause tissue damage, this arm of the immune response needs to be tightly controlled (1). IL-10 is a potent regulatory cytokine produced by a range of immune cells that can suppress inflammation directly via T cell inhibition or indirectly by targeting antigen presenting cell (APC) functions (10). The production of IL-10 by Th1 cells has emerged as an important mechanism for dampening T cell and APC activation in the face of intractable infection, including in patients with visceral leishmaniasis (11), active pulmonary tuberculosis (12), primary HIV infection (13), and malaria (in children in Gambia) (14). These IL-10–producing Th1 (type I regulatory T [Tr1]) cells are distinct from FoxP3-expressing Tregs (15, 16), and their development in mice is driven by IL-27 signaling (17–20) and is dependent on the transcription factors cMaf (21, 22) and Blimp-1 (23–25). Tr1 cells are characterized by high levels of LAG3 and CD49b expression during intestinal inflammation and helminth infection (26). While high levels of PD1, CTLA4, TIM3, CCR5, GITR, granzyme B, TGFβ, ICOS, and IL-12Rβ2 have been reported, the expression of these molecules is heterogeneous across Tr1 cells (27). Furthermore, in addition to including IL-10–producing Th1 cells, Tr1 cells may also include other CD4^+^ T cell subsets that also produce TGFβ and have suppressor functions independent of FoxP3, depending on the inflammatory context (16). Although Tr1 cells protect tissue from inflammation (15, 16), they can also impair disease resolution by suppressing Th1 cell–mediated immunity, thereby allowing for persistence of infection (20, 24).

The rapid engagement of immunoregulatory responses is a key feature of infections that generate inflammation (7). In controlled human malaria infection (CHMI) studies where healthy volunteers have submicroscopic *Plasmodium falciparum* infection, parasite antigen-specific Tr1 cells expand (28). Parasite-specific Tr1 cells also quickly emerge in other parasitic diseases and affect control of parasite growth (20, 24, 29–31). Importantly, Tr1 cells likely influence patient responses to antiparasitic drug treatment because of the need for T cell help to ensure optimum drug efficacy (32, 33). Parasite-specific immunoregulatory networks may also affect vaccine efficacy in disease endemic settings because Tr1 cells, rather than CD4^+^ T cell subsets that promote desired proinflammatory and/or antibody-mediated responses, dominate host immune responses (7, 34). Hence, modulating Tr1 cell development and/or behavior represents a potentially important approach to improving vaccine efficacy. Thus, a better understanding of Tr1 cell development and function may aid in the identification of strategies to manipulate these cells for clinical advantage.

Here, we sought to identify molecular signatures that distinguished IL-10^–^Th1 cells from IL-10^+^Tr1 cells during malarial infection in people participating in CHMI studies with blood-stage *P*. *falciparum*, and in C57BL/6 mice infected with *P*. *berghei* ANKA (*Pb*A). We identified a Tr1 cell phenotype, as well as candidate molecules for immune modulation. Together, these results provide insights into Tr1 cell development and identify molecules for targeting to improve antiparasitic immunity.

## Results

### A Tr1 cell gene signature for human malaria.

**Figure 1:**
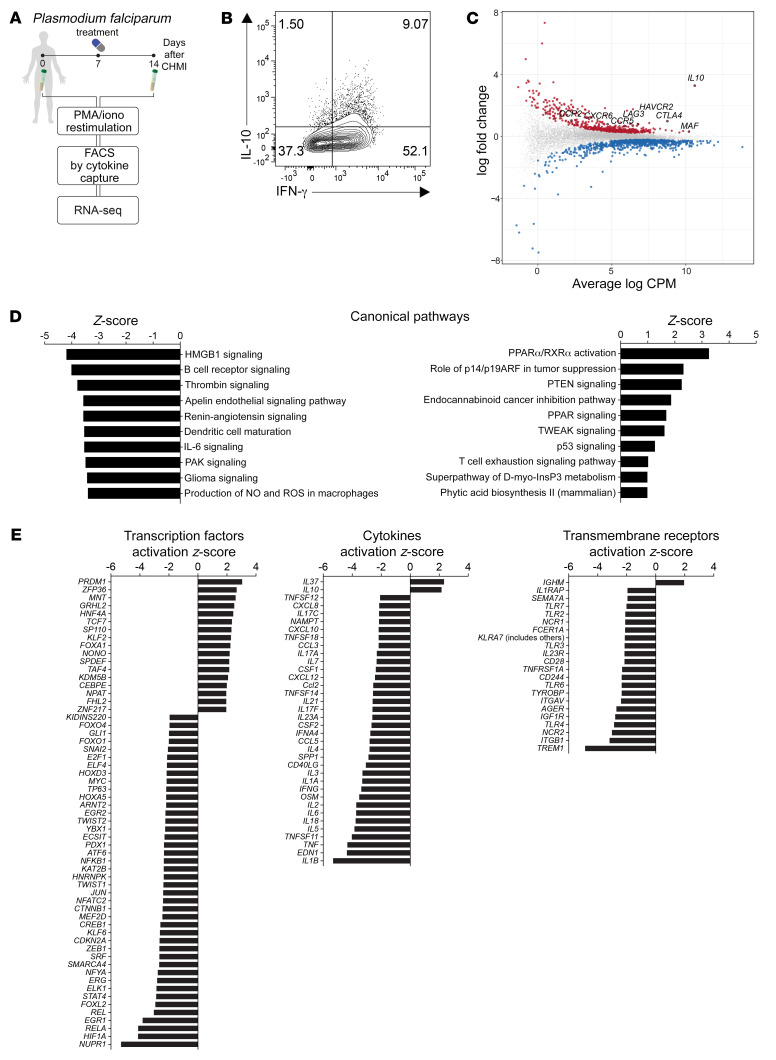
A molecular signature for human Tr1 cells during *P.* *falciparum* malaria. A schematic showing a brief outline of the work flow (**A**) for isolating peripheral blood Th1 (IFN-γ^+^IL-10^–^) and Tr1 cells (IFN-γ^+^IL-10^+^) (**B**). Mean-difference plot from differential expression analysis between Tr1 and Th1 cells (**C**). The 521 upregulated DEGs are colored red, the 794 downregulated DEGs are colored blue, and nonsignificant genes are colored grey. Genes of interest are labelled and outlined in black. (**D**) The top 10 up (right) and downregulated (left) canonical pathways identified in Tr1 cells relative to Th1 cells are listed, as well as significantly different predicted upstream transcription factors, cytokines and transmembrane receptors between the 2 cell populations (**E**). The analysis was performed on 5 paired Th1 and Tr1 cell samples isolated from the blood of volunteers participating in CHMI studies with *P*. *falciparum*.

To identify molecules that demarcate IL-10^–^Th1 cells from IL-10^+^Tr1 cells and potentially contribute to their distinct functions, we first used cytokine capture based on IFN-γ and IL-10 expression following ex vivo stimulation with phorbol ester and calcium ionophore. Th1 (CD4^+^IFN-γ^+^IL-10^–^), Tr1 (CD4^+^IFN-γ^+^IL-10^+^), and cytokine-negative (CD4^+^IFN-γ^–^IL-10^–^) cells were sorted from the blood of CHMI participants infected with blood-stage *P*. *falciparum*, 7 days after antiparasitic drug treatment (14 days p.i.) (Figure 1, A and B). We chose this time point for the peak Tr1 cell responses, which were established previously (28). The isolation of enriched Th1 and Tr1 cells was confirmed (Supplemental Figure 1; supplemental material available online with this article; https://doi.org/10.1172/JCI153733DS1), and RNA-Seq was employed to determine differentially expressed genes (DEGs) between Tr1 and Th1 cells (Figure 1C and Supplemental Table 1). We identified 1,315 DEGs in Tr1 cells compared with Th1 cells, with 521 and 794 of these significantly up- and downregulated, respectively. Upregulated DEGs included those encoding the coinhibitory receptor molecules *CTLA4*, *HAVCR2* (encoding TIM3), and *LAG3*; chemokine receptors *CCR2*, *CCR5*, and *CXCR6*; as well as the transcription factor *MAF* (Figure 1C). Ingenuity pathway analysis of the data set led to identification of major upregulated canonical pathways, including those associated with peroxisome proliferator-activated receptor and T cell exhaustion, while major downregulated canonical pathways included those associated with IL-6 signalling (Figure 1D). Of note, the majority of upstream transcription factors, cytokines, and transmembrane receptors predicted by this analysis were downregulated in Tr1 cells relative to Th1 cells (Figure 1E). Exceptions included *PRDM1* (encoding BLIMP-1), *IL10*, and *IL37*; with *BLIMP1* and *IL10* expression previously being strongly associated with Tr1 cell immunoregulatory functions (16).

**Figure 2:**
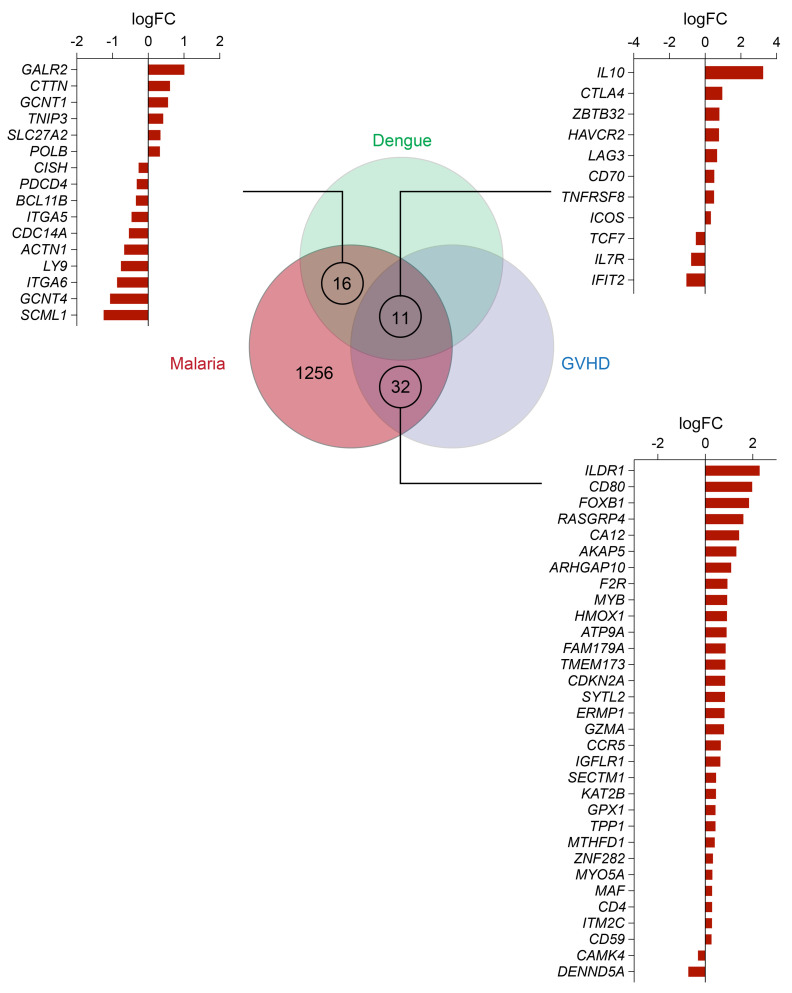
Common DEGs associated with human Tr1 cells in malaria, dengue fever and GVHD. DEGs from malaria Tr1 and Th1 cell comparisons (from Figure 1) were compared to the top 250 genes in IL-10^+^IFN-γ^+^ versus IL-10^–^IFN-γ^+^ dengue virus-specific CD4^+^T cells (ref. 35) and to 289 DEGs in alloantigen-specific Tr1 cells versus non-Tr1 cells (ref. 36). The Venn diagram shows 11 Tr1 cell DEGs were common to all 3 diseases.

To identify a conserved transcriptional signature across different human diseases, we next compared our human Tr1 cell transcriptional signature with 2 others reported from dengue (35) and graft-versus-host disease (GVHD) patients (36) (Figure 2 and Supplemental Table 2). We compared DEGs identified between Tr1 and Th1 cells in CHMI to the top 250 genes in IL-10^+^IFN-γ^+^ versus IL-10^–^IFN-γ^–^ dengue virus–specific CD4^+^ T cells (only the top 250 genes were reported), and to the DEGs in alloantigen-specific Tr1 cells versus other non-Tr1 subsets. Genes that were differentially expressed, based on log fold change (FC) scores, in opposite directions in the 2 data sets were excluded. This analysis resulted in the identification of 8 common DEGs that were upregulated in Tr1 cells from the 3 disease groups — *IL-10*, *CTLA4*, *ZBTB32*, *HAVCR2*, *LAG3*, *CD70*, *TNFRSF8* (encoding CD30), and *ICOS* — as well as 3 common downregulated DEGs — *TCF7*, *IL7R* and *IFIT2*. Another 16 and 32 malaria Tr1 cell DEGs were shared with dengue and GVHD Tr1 cells, respectively. However, there were many different DEGs between Tr1 cells from these patient groups, and although some of these differences may have arisen because the data sets were generated at different times and by different groups, it is also likely that there is heterogeneity in the transcriptional Tr1 cell signatures among diseases, reflecting the development of these cells in different inflammatory contexts.

### A Tr1 cell gene signature for experimental murine malaria.

**Figure 3:**
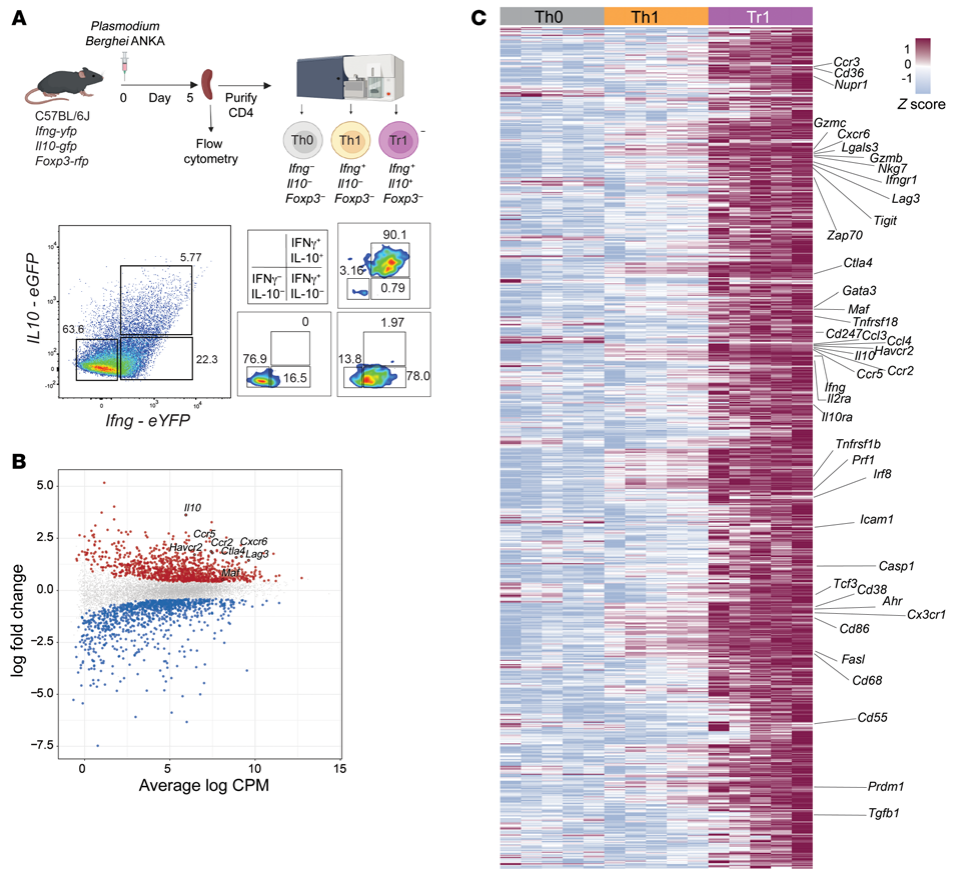
A molecular signature for mouse Tr1 cells during experimental malaria caused by *Pb*A. A schematic showing the workflow for isolating splenic Th0 (FoxP3^–^IFN-γ^–^IL-10^–^), Th1 (FoxP3^–^IFN-γ^+^IL-10^–^), and Tr1 cells (FoxP3^–^IFN-γ^+^IL-10^+^) for RNA-Seq analysis (**A**). Mean-difference plot from differential expression analysis between Tr1 and Th1 cells (**B**). The 1,025 upregulated DEGs are colored red, the 1,006 downregulated DEGs are colored blue, and nonsignificant genes are colored grey. Genes of interest are labelled and outlined in black. A heat map with the top 1,000 upregulated DEGs between Tr1 and Th1 cells is shown with previously identified Tr1 cell-associated gene signatures labelled on the right (**C**). Th0, Th1, and Tr1 cells from the spleens of the same animals (*n* = 5) were compared.

To identify a Tr1 cell gene signature in an experimental malaria model that would allow future functional testing of candidate signature molecules in vivo, we infected triple reporter (*Il10^gfp^* × *Ifng^yfp^* × *foxp3^rfp^*) C57BL/6 mice with *PbA* and then isolated Th0 (CD4^+^GFP^–^YFP^–^RFP^–^), Th1 (CD4^+^GFP^–^YFP^+^RFP^–^), and Tr1 (CD4^+^GFP^+^YFP^+^RFP^–^) cells from the spleens by cell sorting at day 5 p.i. Due to their low numbers, we were unable to obtain sufficient numbers of IL-10–single positive cells (CD4^+^GFP^+^YFP^–^RFP^–^) for further analysis (Figure 3A). RNA-Seq was employed to identify DEGs between the CD4^+^ T cell subsets (Supplemental Table 3). We focused on differences between Tr1 and Th1 cells and found 2031 DEGs in Tr1 cells compared with Th1 cells, with 1,025 and 1,006 of these significantly up- and downregulated, respectively (Figure 3B). Upregulated DEGs included all the coinhibitory receptor molecules common to human Tr1 cells (Figure 2), except for CD70, as well as the chemokine receptors *Ccr2*, *Ccr5*, and *Cxcr6*, and transcription factors *Maf*, *Ahr*, and *Prdm1* (Figure 3C and Supplemental Table 3). Gene ontology (GO) analysis of the DEGs identified biological processes that were enriched, including those associated with T cell activation (Supplemental Figure 2A); Kyoto Encyclopedia of Genes and Genomes (KEGG) pathway analysis of the DEGs identified cytokine-cytokine receptor interaction as a major enriched pathways associated with this conserved mouse Tr1 cell gene signature (Supplemental Figure 2B). Therefore, a core set of coinhibitory receptors, transcription factors, chemokine receptors and effector molecules were common to mouse and human Tr1 cells, as well as other T cell signaling pathways and biological processes that differed between mouse Th1 and Tr1 cells during *Pb*A infection.

### A conserved Tr1 cell signature for Plasmodium infection between mice and humans.

**Figure 4:**
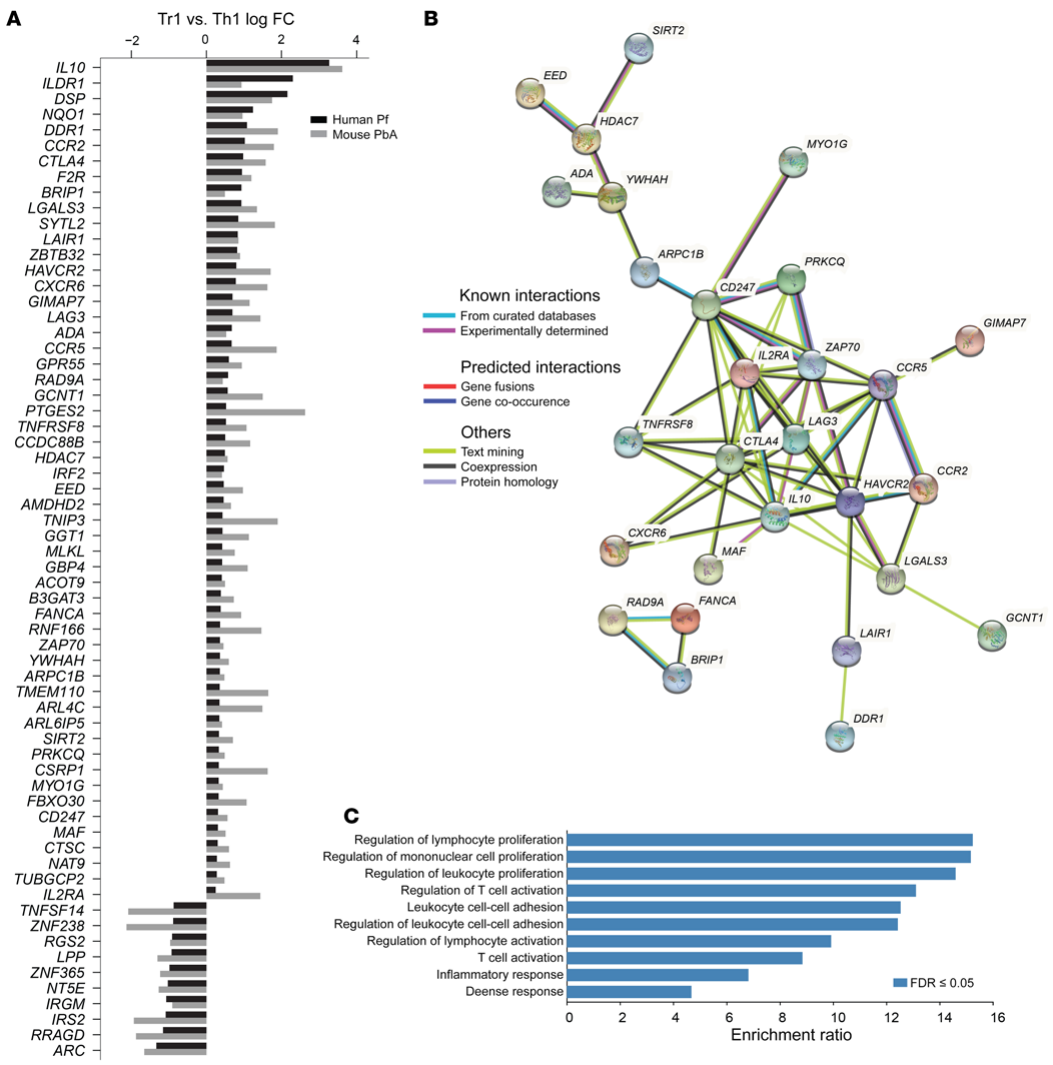
Common DEGs and pathways in human and mouse Tr1 cells relative to Th1 cells during *Plasmodium* infection. All upregulated DEGs and the top 10 downregulated DEGs in Tr1 cells relative to Th1 cells are shown (**A**). The interactions between these genes were assessed using the STRING: protein-protein interaction networks functional enrichment analysis. Genes found with no interactions were removed (**B**). Gene over-representation analysis was used to identify enriched biological processes based on the common DEGs in both human and mouse Tr1 cells, relative to Th1 cells. Selected processes with a positive enrichment ratio are shown in (**C**).

We next sought to identify a Tr1 cell gene signature conserved across host species during *Plasmodium* infection. We converted mouse gene names to human orthologs and excluded any mouse genes for which no human ortholog was identified, as well as noncoding RNA and gene fusions. A total of 54 DEGs were upregulated and 105 DEGs were downregulated in Tr1 cells relative to Th1 cells, and these same genes were shared between humans infected with *P*. *falciparum* and mice infected with *Pb*A (Figure 4A and Supplemental Table 4). These included genes encoding previously identified coinhibitory receptors, such as *CTLA4*, *HAVCR2* (encoding TIM3), and *LAG3*; chemokine receptors, including *CCR2*, *CCR5*, and *CXCR6*; as well as the *MAF*, *IRF2*, and *ZBTB32* transcription factors (Figure 4A). Search Tool for the Retrieval of Interacting Genes/Proteins (STRING): interaction network analysis was employed to predict protein-protein interactions associated with this *Plasmodium* Tr1 cell gene signature. This revealed several predicted interaction networks, including a tight network involving IL-10; the coinhibitory receptors CTLA4, LAG3, and TIM3; and the chemokine receptors CCR2 and CCR5 (Figure 4B). Gene over-representation analysis identified lymphocyte and T cell proliferation and activation as major enriched biological processes associated with this conserved Tr1 cell gene signature (Figure 4C). Thus, we have identified a conserved Tr1 cell gene signature in human *P*. *falciparum* infection and experimental malaria, comprising a number of DEGs with a suite of predicted interactions.

### Expansion of Tr1 cells with increased expression of coinhibitory receptors following Plasmodium falciparum infection in humans.

We next examined cell surface expression of the Tr1 cell-associated coinhibitory and chemokine receptors identified above in people infected with *P*. *falciparum*. However, we first tested whether we could identify Tr1 cells without the need for restimulation with PMA and ionomycin to detect IFN-γ and IL-10, a potentially confounding process that can alter expression of some cytokines, transcription factors, and cell surface molecules. Previous studies have classified Tr1 cells as CD4^+^LAG3^+^CD49b^+^ (excluding Tregs based on CD127 and CD25 expression) (26, 27). Therefore, we used CHMI PBMCs from a volunteer cohort on day 16 p.i. with *P*. *falciparum* (8 days after drug treatment) to test whether these molecules marked antigen-specific Tr1 cells during malaria. Cells were stimulated for 18 hours with parasitized red blood cells (pRBC) to activate *P*. *falciparum*-specific CD4^+^ T cells prior to cell sorting into CD4^+^ T cell subsets based on LAG3 and CD49b expression (excluding Treg cells; Supplemental Figure 3A) and qPCR performed to measure *IFNG* and *IL10* mRNA levels (Supplemental Figure 3B). We found that CD4^+^LAG3^+^CD49b^+^ T cells expressed the highest levels of *IFNG* and *IL10* combined, and therefore used this combination of markers to identify Tr1 cells in the studies below.

**Figure 5:**
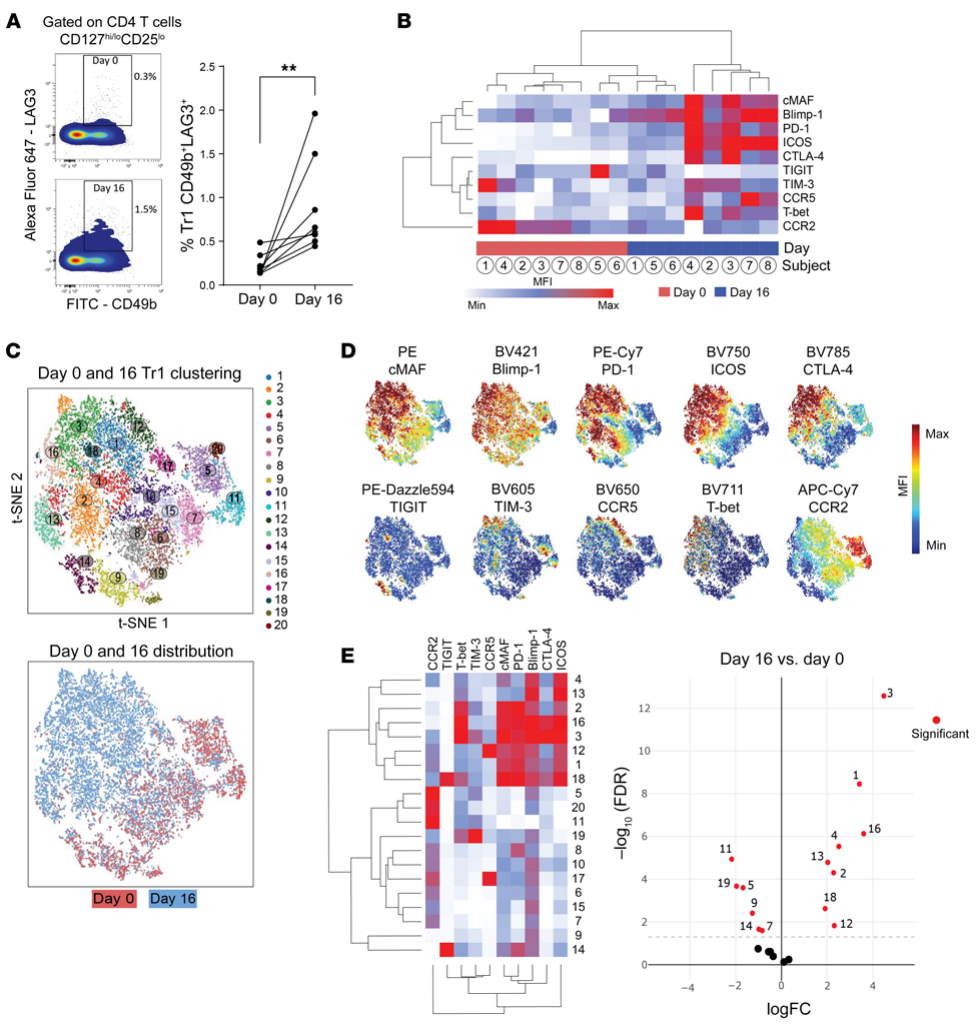
Development of coinhibitory receptor rich Tr1 cells during CHMI studies. Peripheral blood Tr1 cells defined by high levels of CD49b and LAG3 expression were assessed by FACS from volunteers participating in CHMI studies prior to infection (Day 0) and 8 days after antiparasitic drug treatment (Day 16) (**A**). The expression of cMAF, BLIMP-1, PD1, ICOS, CTLA4, TIGIT, TIM3, CCR5, Tbet, and CCR2 on Tr1 cells was measured, and MFI of staining presented as a heat map (**B**). Clustering of Tr1 cells based on MFI of all molecules (**C**) and individual molecule expression in the tSNE plot (**D**) was performed. Coinhibitory–receptor rich clusters among Tr1 cells can be visualized in the heatmap,and clusters that are significantly different between day 0 and 16 after infection are indicated (**E**). Arcsinh-scaled MFI values used to generate heat maps are shown in Supplemental Table 5. *n* = 8 paired volunteer samples; **P < 0.01; significance assessed by Mann-Whitney test (**A**) and using edgeR for all clusters at day 0 and day 16 after infection, indicated by red circles (**E**).

In addition to examining expression of the Tr1 cell-associated coinhibitory and chemokine receptors identified above in humans infected with *P*. *falciparum*, antibodies against PD1 and TIGIT were also included in our flow cytometry panels due to their past associations with Tr1 cells in the context of intestinal inflammation (27) and the known immunomodulatory role for PD1 in malaria (37–39). We also included cMAF and BLIMP-1 because the former was a conserved Tr1 cell DEG (Figure 1C and Figure 4A), and both have been associated with the induction of *Il10* transcription in mice (21, 24, 25, 40). As previously reported (28), a significant increase in the frequency of Tr1 cells was observed in the blood of volunteers participating in CHMI studies with *P*. *falciparum*, 8 days after antiparasitic drug treatment (16 days p.i.; Figure 5A). When Tr1 cells from the same individuals at days 0 and 16 p.i. were compared, the expression of BLIMP-1, CCR5, Tbet, ICOS, cMAF, CTLA4, and PD1, as well as the frequency of Tr1 cells expressing these molecules was significantly increased following infection. However, this was not the case for TIM3, TIGIT, or CCR2 expression (Figure 5B, Supplemental Figure 4, and Supplemental Table 5). The elevated expression of LAG3, cMAF, ICOS, CTLA4, and CCR5 on Tr1 cells at day 16 p.i. was consistent with the above RNA-Seq data (Figure 1C and Supplemental Table 1), further supporting the hypothesis that these molecules serve as Tr1 cell markers during human *P*. *falciparum* infection. However, it should be noted that there was significant heterogeneity in the expression of all molecules investigated between individuals (Figure 5B).

Molecular and functional heterogeneity has been reported among Tr1 cells (27). Thus, the heterogeneity of expression of the above molecules on Tr1 cells was investigated. Using unsupervised clustering of Tr1 cell FACS data from the blood of volunteers participating in CHMI studies, we identified 20 unique clusters associated with infection (Figure 5C). Furthermore, and consistent with a previous report (27), Tr1 cells could be readily classified into subpopulations expressing most coinhibitory receptors (coinhibitory-receptor rich), and those expressing far fewer coinhibitory receptors (coinhibitory-receptor poor) (Figure 5, D and E). The frequency of coinhibitory–receptor rich clusters 1, 2, 3, 4, 12, 13, 16, and 18 increased following infection, while there was a decrease in the frequency of coinhibitory–receptor poor clusters 5, 7, 9, 11, 14, and 19 following infection and drug treatment (Figure 5E). Thus, infection of humans with *P*. *falciparum* was associated with the expansion of Tr1 cells that were coinhibitory-receptor rich.

### Expression of coinhibitory receptor molecules on Tr1 cells during experimental murine malaria.

**Figure 6:**
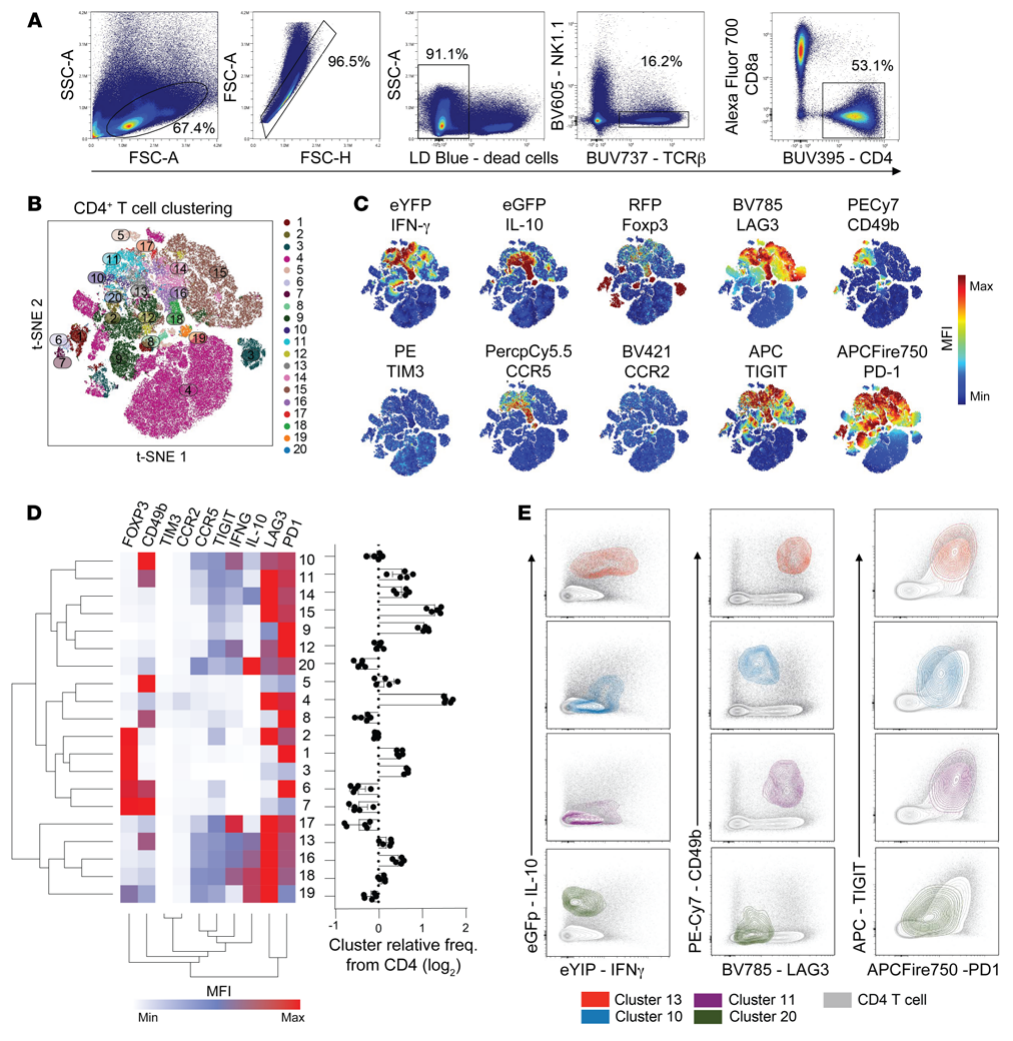
Development of coinhibitory–receptor rich Tr1 cells in the spleen during experimental malaria caused by infection of triple reporter (*Il10gfp* × *Ifngyfp* × *Foxp3rfp*) C57BL/6 mice with *Pb*A. Splenic CD4^+^T cells were identified by flow cytometry at day 5 after infection (**A**). Clustering of cells based on expression of IFN-γ, IL-10, FoxP3, CD49b, LAG3, PD1, TIGIT, TIM3, CCR2, and CCR5 (**B**) and individual molecule expression by tSNE plot (**C**) was performed. The MFI of staining for all 20 cell clusters identified was presented as a heat map, along with the relative frequency of each cluster (**D**). Selected clusters were then overlayed on all CD4^+^T cells and the expression of IL-10 and IFN-γ, LAG3 and CD49b, or PD1 and TIGIT is shown (**E**). *n* = 5 individual mice (**B**–**E**). Arcsinh scaled MFI values used to generate heat maps are shown in Supplemental Table 5.

We next examined coinhibitory receptor expression by splenic CD4^+^ T cells from triple reporter (*Il10^gfp^* x *Ifng^yfp^* x *Foxp3^rfp^*) C57BL/6 mice infected with *Pb*A (Figure 6A). As expected, no Th1 or Tr1 cells, as defined by GFP or YFP expression, were detected in naive mice. Using unsupervised clustering of splenic CD4^+^ T cells isolated on day 5 p.i., we identified 20 unique clusters (Figure 6B). Furthermore, we observed coinhibitory receptor-rich and -poor clusters in the general CD4^+^ T cell population, as well as Tr1 cells defined by either IFN-γ and IL-10 or LAG3 and CD49b coexpression (Figure 6, C and D, and Supplemental Table 5). Cluster 13 comprised CD4^+^ T cells that were FoxP3^–^ and coexpressed IFN-γ and IL-10, as well as LAG3 and CD49b, similar to clusters 10, 11 and 20, although cluster 20 produced substantially more IL-10 than IFN-γ (Figure 6E). It should be noted that LAG3 and CD49b coexpression did not define IL-10^+^Tr1 cells in mice as clearly as in humans. Regardless, these clusters also expressed relatively high levels of all of the other molecules examined, with the exceptions of FoxP3, TIM3, and CCR2, with the latter 2 molecules only being expressed by cluster 4. Thus, consistent with observations from people infected with *P*. *falciparum*, we found significant expression of coinhibitory receptors on Tr1 cells, relative to other CD4^+^ T cell subsets, as well as substantial heterogeneity in expression of these molecules during experimental murine malaria caused by *Pb*A infection.

### The roles of PRDM1 and cMAF in human CD4^+^ T cells.

**Figure 7:**
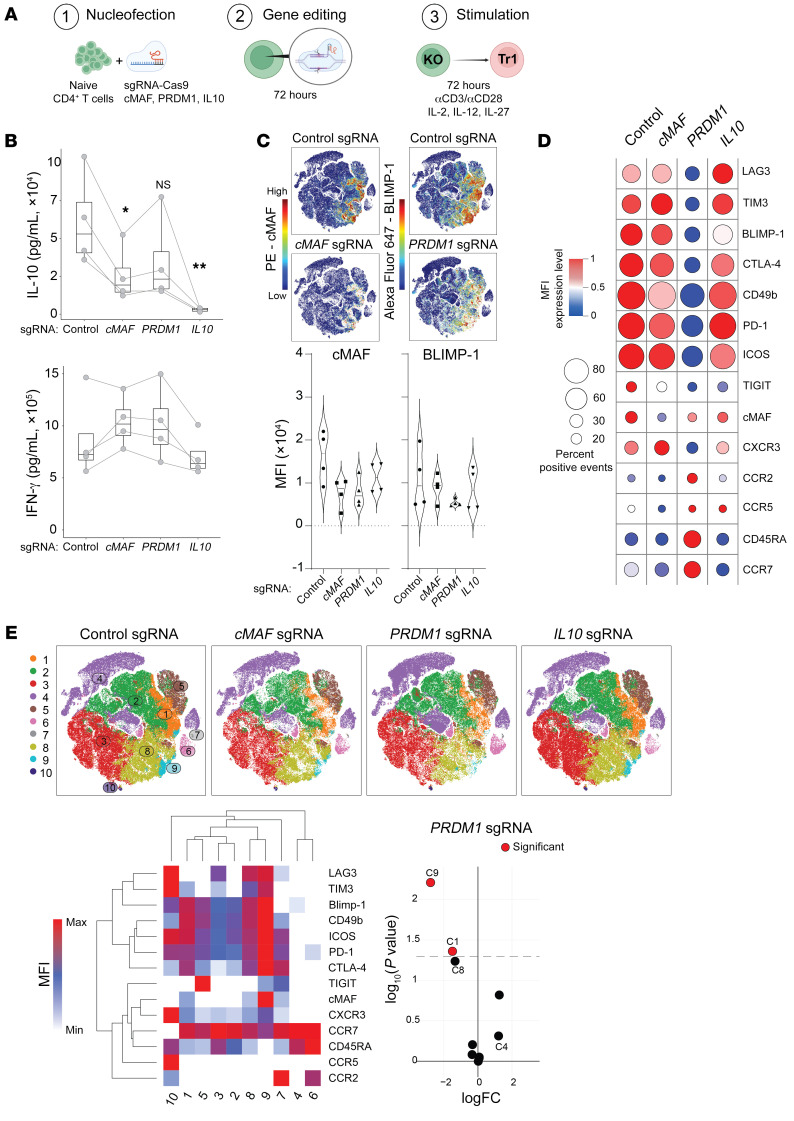
Distinct roles for cMAF and BLIMP-1 in human Tr1 cell development and functions. CRISPR/Cas9 editing of *cMAF*, *PRDM1*, and *IL10* was conducted in primary human CD4^+^T cells, followed by activation and assessment (**A**). IL-10 and IFN-γ levels were measured in cell culture supernatants after 72 hours of activation (**B**). The expression of cMAF and BLIMP-1 is shown in tSNE plots, as well as in violin plots for each sgRNA-treated group (**C**). The expression (as MFI) and percentage (as positive events) is summarized in dot-plot format (**D**). Clustering based on the MFI of all molecules in each treatment group (upper tSNE) and individual molecules (lower heat map) was performed, and clusters significantly different between control and *PRDM1* sgRNA groups are indicated by red circles (**E**). *n* = 4 paired volunteer samples in each treatment group; *P < 0.05 and **P < 0.01 were assessed by paired 1-way ANOVA (**B**) and significance was assessed using edgeR for clusters, indicated by red circles (**E**). Arcsinh scaled MFI values used to generate heat maps are shown in Supplemental Table 5.

Important roles for the transcription factors Blimp-1 and cMaf for IL-10 production and expression of coinhibitory receptors by CD4^+^ T cells in mice have been reported previously (24, 25, 40–42). However, their roles in human CD4^+^ T cells is less clear. Therefore, we employed CRISPR/Cas9 to inactivate the genes encoding these molecules in human primary CD4^+^ T cells (Figure 7A), as previously described (43). We then examined the effect of gene disruption on IL-10 production and expression of coinhibitory receptors by CD4^+^ T cells. We also inactivated the *IL10* gene to establish whether IL-10 contributed directly to any of these activities. To promote Tr1 cell development, we first optimized cell culture conditions to induce IFN-γ, IL-10, and Tr1 cell-associated coinhibitory receptor expression by primary human CD4^+^ T cells (Supplemental Figure 5A). We found IL-12 and IL-27 in combination augmented both IL-10 and IFN-γ production (Supplemental Figure 5B), as well as the expression of multiple coinhibitory receptors (Supplemental Figure 5, C and D). Therefore, following gene editing, we activated CD4^+^ T cells by stimulation with anti-CD3ε and anti-CD28 mAbs in the presence of IL-2, IL-12, and IL-27. After CD4^+^ T cell activation, *IL10* inactivation resulted in minimal IL-10 production, but had limited impact on IFN-γ production (Figure 7B), suggesting a limited autocrine or paracrine role for IL-10 in influencing the strength of CD4^+^ T cell responses. We also confirmed reduced cMAF and BLIMP-1 expression following *cMAF* and *PRDM1* inactivation, respectively, although inhibition of expression of these molecules was not complete (Figure 7, C and D, and Supplemental Figure 6A), with cMAF deletion in around 70% and BLIMP-1 deletion in about 40% of CD4^+^ T cells after 72 hours of stimulation (Figure 7C and Supplemental Figure 6, B and C). Nevertheless, *cMAF* inactivation caused significantly reduced IL-10 production, and although *PRDM1* editing lowered IL-10 levels, this did not reach statistical significance. Again, we found no significant effect on IFN-γ production with any gene editing (Figure 7B). However, only *PRDM1* inactivation resulted in significant reductions in the expression of coinhibitory receptors and other molecules associated with the Tr1 cell signature we identified above on CD4^+^ T cells after 72 hours of culture (Figure 7, D and E, Supplemental Figure 6, and Supplemental Table 5). This was despite *PRDM1* gene editing being the least efficient of all genes modified (Figure 7C). It should be noted that this analysis was performed on all CD4^+^ T cells in culture because the majority of Tr1 cells that developed after stimulation derived from cells that had not been gene edited. Using unsupervised clustering of CD4^+^ T cell FACS data collected following gene editing and cell culture, we identified 10 unique clusters associated with CD4^+^ T cells under Tr1 cell culture conditions (Figure 7E). Again, we found heterogeneity in coinhibitory receptor expression with, for example, clusters 9 and 4 being coinhibitory receptor rich and poor, respectively (Figure 7E). However, significant changes in cluster distribution only occurred in *PRDM1*-edited CD4^+^ T cells, including reduced expression of coinhibitory receptor–rich cluster 9 (Figure 7E). Thus, our results show important but distinct roles for cMAF and BLIMP-1 in induction of IL-10 and coinhibitory receptor expression by human CD4^+^ T cells, respectively.

## Discussion

In this study, we identified a molecular signature that distinguished human IL-10^–^Th1 cells from IL-10^+^Tr1 cells in malaria — a parasitic disease with high morbidity, yet unmet medical needs. We also report a conserved human Tr1 cell transcriptional signature associated with the disparate diseases GVHD, dengue fever, and malaria. Tr1 cells from all diseases expressed many coinhibitory receptors and other immune regulatory molecules, but we found significant heterogeneity in types of receptors and levels of expression by Tr1 cells that has implications for the development of strategies aimed at targeting these molecules for clinical advantage. We also showed that while cMAF played an important role in IL-10 induction, BLIMP-1 was required for development of coinhibitory receptor–rich human Tr1 cells.

Tr1 cells are enigmatic contributors to host immune responses. They play a critical role in limiting tissue pathology caused by infection through their antiinflammatory functions (15, 16, 26). However, it is via this process that they can also allow pathogens to persist, thereby maintaining or even exacerbating ensuing disease (24). Tr1 cells emerge rapidly after first exposure to pathogens (11, 14, 28, 31, 34, 44, 45), with a profound influence on disease outcome, which also dictates cellular responses to subsequent infections by the same pathogen (7). Although previous work has outlined the differentiation of this population from effector CD4^+^ T cell subsets such as Th1 cells (46), there are still large gaps in our knowledge about Tr1 cell development and maintenance. A recent study suggested that Tr1 cells represent a phenotypically and functionally heterogeneous cell population (27). Our data supports this, whereby we find both coinhibitory receptor rich and poor subpopulations among Tr1 cells from mice infected with *Pb*A and humans infected with *P*. *falciparum*. These findings have clear implications for the therapeutic potential of coinhibitory receptor blockade and help to explain the heterogeneity of individual responses as well as the improved clinical responses to combinations of coinhibitory receptor blocking measures.

Type I IFNs regulate the expression of coinhibitory receptors in human T cells, and computational approaches identified the transcription factor *SP140* as a key regulator in this network (47). We previously reported an important role for type I IFNs in the development of human Tr1 cells during malaria (24), but did not identify *SP140* as a DEG in our comparison of human Th1 and Tr1 cells. However, *SP140* was increasingly expressed by both CD4^+^ T cell subsets compared with cytokine-negative CD4^+^ T cells, but it was not significantly different in any of our comparisons of mouse CD4^+^ T cell subsets, possibly reflecting a species difference in this cytokine-signalling pathway. We did find that *cMAF* was a DEG distinguishing Tr1 and Th1 cells, and that *PRDM1* was predicted to be an important upstream transcription factor for human Tr1 cell development. *cMaf* and *Prdm1* have previously been shown to form part of a coinhibitory receptor–rich Tr1 cell transcriptional signature in mice with experimental colitis (27). In experimental tumour models, *cMaf* and *Prdm1* were identified as cooperative regulators of coinhibitory receptor expression by T cells (41). cMaf was also reported to be important for the induction of CD4^+^ T cell-produced IL-10, which is required to suppress Th1-, Th2-, and Th17-mediated pathology in experimental models of malaria, allergy, and autoimmunity, respectively (40). Our results provide evidence for similar roles in human CD4^+^ T cells. However, we also identified differences in the roles of cMAF and BLIMP-1. For example, while the absence of cMAF had a greater effect on IL-10 production, the absence of BLIMP-1 had a more profound affect on coinhibitory receptor expression, and in particular, the emergence of coinhibitory–receptor rich Tr1 cells. Thus, a better understanding of the roles and interactions of transcription factors on human CD4^+^ T cell subset development will be important to better harness their therapeutic potential to improve disease outcomes.

We and others have previously determined that Tr1 cells emerge early after infection and play important roles in protecting tissue from inflammation, yet suppress antiparasitic immunity (11, 24, 28). This has important implications for future responses to vaccination, whereby parasite antigens stimulate potent Tr1, but not antiparasitic Th1 or Tfh cell responses (7). It is notable that Tr1 cell responses in children living in malaria-endemic areas dominate recall responses to *Plasmodium* antigens (14, 34, 44, 45) while, in the same populations, malaria vaccine efficacy is relatively poor (48). Despite the RTS,S malaria vaccine showing a protective efficacy of around 50% in healthy malaria-naive individuals residing in the USA (49), vaccine efficacy fell to around 30% in both adults and children living in malaria endemic regions (48). Similar results for an irradiated sporozoite vaccine delivered intravenously, albeit with more dramatic differences in vaccine efficacy, have been reported following vaccination. High levels of protection (~70%) were reported among healthy malaria-naive individuals residing in the USA (50), while relatively low levels (~20%–30%) of protection were induced in preexposed adults living in malaria endemic areas (51, 52). Therefore, if Tr1 cell responses impede vaccine efficacy, then their transient modulation at the time of vaccination by the inclusion of appropriate agents in vaccine formulations may help to open the bottleneck currently preventing the implementation of efficacious vaccines for parasitic diseases such as malaria. Similarly, manipulating Tr1 cells in the context of drug treatment may represent another approach to improving antiparasitic immunity following natural infection in people living in malaria endemic areas. The molecules identified in our studies represent potential targets for Tr1 cell manipulation.

There are several potential limitations in our study, including our inability to determine whether Tr1 cells form stable memory pools or have a transient existence following *Plasmodium* infection. Furthermore, whether Tr1 cells contribute to parasite persistence in individuals living in malaria-endemic areas has yet to be established. Similarly, the effect of preexisting parasite-specific Tr1 cells on vaccine and/or drug efficacy needs to be determined if they are to be targeted to improve protective immunity against malaria in disease-endemic areas.

In summary, we demonstrated that Tr1 cells in malaria comprise heterogeneous cell populations, based on their expression of coinhibitory receptors, chemokine receptors and transcription factors. Furthermore, we identified unique molecular signatures that distinguish these important regulatory cells from the effector Th1 cell population they derive. We can use this information to identify appropriate molecular targets to manipulate Tr1 cells not only in parasitic disease but also in other conditions where these cells influence disease outcome.

## Methods

### Human Plasmodium falciparum infections.

Healthy individuals, aged 18–55 years with no prior exposure to malaria, underwent controlled infection with *P*. *falciparum* (clone 3D7), as part of a series of clinical trials testing the efficacy of various antimalarial drugs in early stage malaria (Supplemental Table 6). *P*. *falciparum* infected RBCs (approximately 1,800 in QP13C05 and QP14C11, and approximately 2,800 for all other cohorts), were injected i.v. into individuals at day 0, and from day 4 postinfection (p.i.) onward, blood parasite burden was measured twice daily by qPCR (53). Additionally, the trial drug was administered 7 or 8 days p.i. when the cut off for treatment was reached (i.e., there were at least 5,000 parasites/mL). Blood was collected on days 0, 7 and 14–16 p.i. into lithium heparin tubes for cellular processing and analysis.

### Processing human blood.

PBMCs were isolated from human blood samples by Ficoll-Paque (GE Healthcare Life Sciences) gradient centrifugation. In some cases CD4^+^ cells were isolated from PBMCs by MACS purification using CD4 (L3T4) MicroBeads (Miltenyi Biotec) according to the manufacturer’s guidelines. PBMCs were stored at –80°C in 10% DMSO / 90% FCS prior to analysis.

### Mice.

Female mice between 6–12 weeks of age were used for all experiments. Mice were grouphoused with a maximum of 6 mice per cage and maintained under pathogen-free conditions at the QIMR Berghofer Medical Research Institute Animal Facility (Herston QLD, Australia). C57BL/6J (RRID: IMSR_JAX:000664) mice were sourced from the Walter and Eliza Hall Medical Research Institute (Kew VIC, Australia). All other mice were bred in house, including C57BL/6*-Foxp3^tm1flv^*/J (*Foxp3^rfp^*; JAX:008374) (54), C57BL/6-*Il10^tm1Flv^*/J (*Il10^gfp^*; JAX:008379) (55), and B6.129S4-*Ifng^tm3.1Lky^*/J (*Ifng^yfp^*, RRID: IMSR_JAX: 017581) mice (56) .

### PbA infections in mice.

Transgenic *Pb*A (231c11; in house laboratory stock, frozen at –80°C) expressing luciferase and GFP under the control of the ef1-α promoter (57), were thawed and injected i.p. into a passage mouse. The passage mouse was sacrificed when blood parasitemia was between 1% and 3% pRBC. Blood was collected and a parasite inoculum containing 5 × 10^5^ pRBC/mL was prepared and mice were injected with 200 μL of the inoculum i.v. (for a total of 1 × 10^5^ pRBC per mouse).

*Pb*A-infected mice were scored daily beginning on day 4 p.i., for symptoms of experimental cerebral malaria, including hunching in posture, piloerection, lethargy, and wobbly gait. Mice were sacrificed at day 5 p.i. by cervical dislocation, and spleen mononuclear cells were isolated and prepared as previously described (24).

### Cell sorting.

For RNA-Seq, PBMCs isolated from volunteers participating in CHMI studies on day 14 p.i. were restimulated with the following at 37°C for 4 hours: Phorbol 12-myristate 13-acetate (50 ng/mL, Sigma-Aldrich) and Ionomycin calcium salt (1 μg/mL, Sigma-Aldrich) in complete RPMI (human) 10% (v/v) FBS (Thermo Fisher Scientific), 20 μg/mL gentamycin (Sigma-Aldrich), and RPMI1640 (Thermo Fisher Scientific) (mouse) 10% (v/v) FBS, 1% (v/v) penicillin/streptomycin (Thermo Fisher Scientific). IL-10 and IFN-γ cytokines were then captured on the surface of the cells using the Miltenyi Biotec IL-10 (PE) and IFN-γ (APC or FITC) Secretion Assays, according to the manufacturer’s guidelines. Cells were then stained with antibodies for 20 minutes at room temperature while protected from light. IL-10^–^IFN-γ^–^,IL-10^–^IFN-γ^+^, and IL-10^+^IFN-γ^+^ cells were sorted from CD19^–^CD16^–^CD14^–^CD8^–^CD4^+^ human cells with a BD FACSARIA III (BD Biosciences). Cells were stored at –80°C in 1% (v/v) 2-mercaptoethanol (Sigma-Aldrich) in RLT buffer (Qiagen).

For qPCR, PBMCs isolated from volunteers participating in CHMI studies on day 16 p.i. (*n* = 8) were restimulated for 18 hours with *P*. *falciparum* pRBC as part of an activation-induced marker assay, as previously described (58). Cells were then stained with antibodies reactive against human CD3ε and CD4 (to identify CD4^+^ T cells), CD25 and CD127 (to exclude regulatory T cells), and LAG3 and CD49b, as described in the Flow Cytometry section below. Conventional CD4^+^ T cells were sorted into LAG3^–^CD49b^–^, LAG3^–^CD49b^+^, LAG3^+^CD49b^–^, and LAG3^+^CD49b^+^, then stored at –80°C in 1% (v/v) 2-mercaptoethanol (Sigma-Aldrich) in RLT buffer (Qiagen).

Mouse splenocytes were isolated on day 5 p.i. after infection with *Pb*A, and CD4^+^ T cells were enriched using MACS negative selection, according to the manufacturer’s instructions (Miltenyi Biotec). Cells were stained for CD4 and CD90.2, plus Zombie Aqua, as described in the Flow Cytometry section below. CD4^+^ T cells were sorted based on RFP-negative (to remove FoxP3^+^CD4^+^ T cells), YFP^+^GFP^–^ (IFN-γ^+^IL-10^–^ [Th1]), YFP^+^GFP^+^ (IFN-γ^+^IL-10^+^ [Tr1]), and YFP^–^GFP^–^ (IFN-γ^–^IL-10^–^ [Th0]), with a BD FACSAria III (BD Biosciences). Cells were stored at –80°C in 1% (v/v) 2-mercaptoethanol (Sigma-Aldrich) in RLT buffer (Qiagen).

### RNA isolation.

RNA was isolated from cells using RNeasy Mini Kit (for human cells), RNeasy Micro Kit (for mouse cells), and Qiashredders as per manufacturer’s instructions (Qiagen). The Nanodrop ND-1000 UV-Vis Spectrophotometer (Thermo Fisher Scientific) was used to determine RNA concentration and quality.

### RNA-Seq.

RNA isolated from human naive, Tr1, and Th1 sorted cells (*n* = 5) was used to synthesize cDNA using the High-Capacity cDNA Reverse Transcription kit (Applied Biosciences) according to manufacturer’s guidelines. RNA was isolated from mouse Th0, Th1, and Tr1 cells and used to synthesize cDNA. Libraries were prepared from cDNA using New England Biolabs Single cell/low input library preparation kit for Illumina, according to the manufacturer’s instructions. 50 bp single-end mRNA-Seq was performed on the Illumina HiSeq (Illumina) by the Australian Genome Research Facility (AGRF, VIC, Australia). RNA-Seq data was then processed using the Galaxy platform (https://galaxy-qld.genome.edu.au/galaxy/) (59). *FastQC* was used for quality control of data (https://www.bioinformatics.babraham.ac.uk/projects/fastqc/), and reads were mapped to the human (GRCh38/hg38) genome using the *STAR* aligner (60). Transcripts were then assembled, and reads per kb of transcript per million mapped reads were estimated using *Cufflinks* (61). *Cuffmerge* was used to merge transcript assemblies, and *HTseq* was used to transform mapped reads into counts based on the GENCODE vM9 annotation for mouse and GENCODE release 24 for human (62–64). Finally, *EdgeR* was used to analyze these counts for differential gene expression (65). The glmFit() function was used to fit a negative binomial generalized log-linear model to the read counts for each gene. Using the glmLRT() function, we conducted genewise–likelihood ratio tests for differential expression between Tr1 and Th1 cells on day 14 p.i. DEGs were determined using a FDR of less than 0.05.

RNA isolated from mouse splenic Th0 (IFN-γ^–^IL-10^–^), Th1 (IFN-γ^+^IL-10^–^), and Tr1 (IFN-γ^+^IL-10^+^) cells (*n* = 5 paired samples) was assessed using the RNA 6000 Pico Kit (Agilent Technologies). The cDNA libraries were prepared using NEBNext Single Cell/Low Input RNA Library Prep Kit for Illumina 96 reactions (New England Biolabs). Libraries were quantified using the KAPA Library Quantification Kit (Roche) and subsequently sequenced on the Illumina NextSeq550 platform (Illumina) as a paired-end 75 cycle run. Approximately 12–32 million paired reads were obtained per sample. Sequence reads were trimmed for adapter sequences using Cutadapt (66) version 1.9 and aligned using STAR (60) version 2.5.2a to the *Mus musculus* GRCm38 assembly with the gene, transcript, and exon features of Ensembl (release 70) gene model. Quality control metrics were computed using RNA-SeqC (67) version 1.1.8 and expression was estimated using RSEM (68) version 1.2.30. All downstream RNA-Seq analysis was performed using R version 3.6.2. Differential expression analysis was performed using the quasi-likelihood pipeline from edgeR version 3.28.0 (65, 69). Specifically, only protein-coding genes that passed the minimum expression filter using edgeR’s filterByExpr function with default settings were kept for further analysis. The design matrix was formed using an additive model formula. Specifically, we used model.matrix (~Subject + Group), where the subject was 1 of 5 donor mice, and the group was Th0, Th1 or Tr1. The glmQLFit function (70) was used to fit a quasi-likelihood negative binomial generalized log-linear model to the read counts for each gene. Using the glmTreat function (71), we then tested for differential expression between Tr1 (YFP^+^GFP^+^; IFN-γ^+^IL-10^+^) and Th1 (YFP^+^GFP^–^; IFN-γ^+^IL-10^–^), cells at day 5 p.i., relative to a minimum fold change threshold of log2(1.2). DEGs were determined using a FDR of less than 0.05 using p.adjust method for “fdr”.

The mean-difference plots (log2 fold change versus average log2 counts per million) were created using ggplot2 (version 3.3.2) based on the respective differential expression analysis results from edgeR.

To compare Tr1 signature genes between mice and humans, mouse Tr1 signature genes (from Figure 3) were first converted to human orthologs using Ensembl BioMart (release 107) (72). We excluded any mouse genes for which no human ortholog was identified, as well as noncoding RNA, and gene fusions, and if 2 or more mouse genes mapped to the same human gene, duplicates were removed. The Venndetail package (version 1.10.0) (https://github.com/guokai8/VennDetail; commit ID 675081b) was used to check for overlaps between Tr1 signatures between human and mouse in the same direction (upregulated versus upregulated; downregulated versus downregulated).

### Pathway and STRING analysis.

Pathway analysis was performed using Ingenuity Pathway Analysis software (Qiagen; build version: 389077M, content version: 27821452 [release date: 2016-06-14]). STRING analysis was performed on https://string-db.org/ (version 11.5). The list of genes within the conserved Tr1 signature was used as an input, with all parameters set at default (network type: full network; meaning of network edges: evidence; minimum required interaction score: medium confidence [0.400]). GO analysis was performed to identify biological processes associated with the DEGs, and KEGG pathway analysis was performed to identify KEGG categories associated with the DEGs. Analysis of GO terms and KEGG categories was performed using several functions from clusterProfiler version 3.14.3 (73). First, the bitr function converted gene IDs of the DEGs from Ensembl to Entrez. Entrez IDs were passed to the enrichGO function with the subontology “BP” or enrichKEGG function, before plotting the results with the dotplot function, exported from enrichplot version 1.6.1. Overrepresentation analysis was performed using WEB-based GEne SeT analysis tool kit (74).

### Comparing Tr1 cell transcriptional signatures from malaria, dengue, and GVHD patients.

DEGs from the Tr1 versus T_Eff_ comparison were compared with the top 250 genes in IL-10^+^IFN-γ^+^ versus IL-10^–^IFN-γ^–^ dengue virus-specific CD4^+^ T cell comparison (35), and to 289 DEGs in alloantigen-specific Tr1 cells versus other non-Tr1 subset comparison (36). Genes that were differentially expressed in 2 data sets but in opposing directions based on logFC were excluded. The results were illustrated as a Venn diagram produced using the ‘venn’ package (v1.10; Dusa, A. 2021, https://CRAN.R-project.org/package=venn) run on R (v4.1.2) and modified on Adobe Illustrator (v26.0.3).

### Human CD4^+^ T cell polarization.

PBMCs were isolated from healthy volunteers. CD4^+^ T cells were isolated using a human CD4^+^ T cell isolation kit (STEMCELL Technologies), according to manufacturer’s guidelines. CD4^+^ T cells (numbering 1 × 10^6^) were then cultured with soluble αCD28 (5 μg/mL, clone CD28.2, BioLegend) and plate bound αCD3ε (wells coated with 1 μg/mL for 4 hours at 37°C, 5% CO_2_, clone UCHT1, BioLegend), supplemented with 50 IU/mL IL-2 (Miltenyi Biotech), 10 ng/mL IL-12 (BioLegend), 100 ng/mL IL-27 (Thermo Fisher Scientific) cell polarizing cytokines in a 48-well plate (for a final volume of 500 μL). After 3 days, cell culture supernatants were collected and stored at –20°C, and cells were assessed by flow cytometry.

### Flow cytometry.

In addition to sorting, flow cytometry was also used to assess fixed cells. Cells were stained with a primary surface stain for 30 minutes at 37°C, 5% CO_2_. After primary staining, cells were washed and cell membranes were then permeabilized using Permeabilization Reagent (eBioscience) for 20 minutes at 4°C. Cells were then stained with an intracellular stain for 45 minutes at 37°C, 5% CO_2_. All surface stains were made up in FACS Buffer, and all intracellular stains were made up in Perm/Wash Buffer (eBioscience). Samples were acquired immediately as described below.

Mouse antibodies used include PerCP/Cy5.5 anti-CCR5 (clone HM-CCR5; BioLegend, 107016), PE anti–TIM-3 (clone B8.2C12; BioLegend, 134004), AF700 anti-CD8α (clone 53-6.7; BioLegend, 100730), APC anti-CD4 (clone GK1.5; BioLegend, 100412), APC anti-TIGIT (clone Vstm3; BioLegend, 142106), BV605 anti-NK1.1 (clone PK136; BD Biosciences, 563220), APCFire 750 anti-CD279 (PD-1, clone 29F.1A12; BioLegend, 135240), BV421 anti-CD192 (CCR2, clone SA203G11; BioLegend, 150605), BV785 anti-CD223 (LAG-3, clone C9B7W; BioLegend, 125219), BUV395 anti-CD4 (clone GK1.5; BD Biosciences, 563790), BUV737 anti-TCRβ (clone H57-597; BD Biosciences, 612821), and PECy7 anti-CD49b (clone HMα2; BioLegend, 103518).

Human antibodies used include FITC anti-CD49b (clone eBioY418; eBioscience, 11049842), BUV496 anti-CD16 (clone 3G8; BD Biosciences, 612945), PE/Fire640 anti-CD19 (clone HIB19; BioLegend, 302274), BUV805 anti-CD14 (clone HCD14; BD Biosciences, 612903), Alexa Fluor 647 anti–LAG-3 (clone 11C3C65; BioLegend, 369304), BV785 anti–LAG-3 (clone 11C3C65; BioLegend, 369322), PerCP/Cy5.5 anti-CD8α (clone RPA-T8; BioLegend, 301032), PerCP/Cy5.5 anti-CD4 (clone OKT4; BioLegend, 317428), PerCP/Cy5.5 anti-CCR7 (clone G043H7; BioLegend, 353220), PerCP/Cy5.5 anti-CD56 (clone HCD56; BioLegend, 318322), APC anti-CD8α (clone RPA-T8; BioLegend, 301049); R718 anti-FOXP3 (clone 259D/C7; BD Biosciences, 566935), BUV395 anti-CXCR3 (anti-CD183, clone 1C6/CXCR3; BD Biosciences, 565223), APC/Cy7 anti-CCR2 (anti-CD192, clone K036C2; BioLegend, 357220), BUV737 anti-CD4 (clone SK3; BD Biosciences, 564305), BV421 anti-CXCR5 (clone RF8B2; BD Biosciences, 562747), BV421 anti-CD56 (clone NCAM16.2; BD Biosciences, 562751), BV421 anti-CD183 (CXCR3, clone 1C6/CXCR3; BD Biosciences, 562558), BV605 anti–TIM-3 (anti-CD366, clone F38-2E2; BioLegend, 345018), BV605 anti-CD4 (clone RPA-T4; BD Biosciences, 562658), BV650 anti-CCR6 (clone 11A9; BD Biosciences, 563922), BV650 anti-CD195 (CCR5, clone 3A9; BD Biosciences, 564999), BV786 anti–CTLA-4 (anti-CD152, clone BNI3; BD Biosciences, 563931), BV421 anti–CTLA-4 (anti-CD152, clone BNI3; BioLegend, 369606), BUV395 anti-CD3ε (clone UCHT1; BD Biosciences, 563540), BUV563 anti-CD45RA (clone HI100; BD Biosciences, 565702), PE anti–c-MAF (clone sym0F1; eBioscience, 12985542), PE/Dazzle TIGIT (clone A15153G; BioLegend, 372715), PE/Cy7 anti–PD-1 (anti-279, clone EH12; BD Biosciences, 561272), PE/Cy7 anti-CD45RA (clone HI100; BD Biosciences, 560675), PE/Cy7 anti-CCR7 (clone G043H7; BioLegend, 353226), BV711 anti-Tbet (clone O4-46; BD Biosciences, 563320), BV750 anti-ICOS (clone C398.4A; BioLegend, 313558), PE/Fire700 anti-CD25 (clone M-A251; BioLegend, 356146), and Alexa Fluor 647 anti–BLIMP-1 (clone 6D3; BD Biosciences, 565002). The Blue Fixable Viability Kit (Thermo Fisher Scientific) or Zombie Aqua (BioLegend) was used to assess cell viability.

### Cytokine analysis.

Cytokine levels were assessed using the BD Cytometric Bead Array (CBA) Human Th1/Th2/Th17 Cytokine Kit (BD Biosciences) as per manufacturer’s instructions. CBA data was analyzed using the FCAP Array Software v3.0 (BD Biosciences).

### Nucleofection.

CRISPR Cas9 gene editing was performed on nonstimulated human CD4^+^ T cells as described previously (43) with some modifications. Briefly, 2 μL sgRNA (120 μM) (Synthego) and 2 μL Cas9-NLS (40 μM) (QB3 Macrolab) were mixed and incubated for 15 minutes at 37°C to make a 3:1 ratio Cas9-RNP complex. Guides used for each gene were *MAF:* GAAGUCAUUAACAUAUUCCA; *IL10:* CAGACAAGGCUUGGCAACCC; *PRDM1:* multi guides, GUGGUGAAGCUCCCUC, UCCCCGGGAGCAAAACC, GGCAGGGAUGGGCUUGG; and control sgRNA #1 (Synthego). Bulk CD4^+^ T cells were purified from cryopreserved PBMCs with the Human CD4^+^ T cell isolation kit (STEMCELL technologies). After purification, cells were counted, washed twice in d-PBS (Thermo Fisher Scientific), and diluted to a concentration of 7.5 × 10^6^ cells in 100 μL of P2 buffer (Amaxa^tm^ P2 primary cell 96-well Nucleofector^tm^ kit, Lonza). Immediately, 4 μL of Cas9-RNP complex was mixed with 20 μL of cells and transferred into a 16-strip nucleovette (Lonza), placed on Amaxa Nucleofector and 96 well Shuttle (Lonza) and treated with the program EH100. After electroporation, 180 μL of warm media was added and cells were transferred to a 96-well plate (Falcon) and incubated for 72 hours at 37°C 5% CO_2_. For CD4^+^ T cell stimulation, 48 flat-bottom plates (Falcon) were coated with 1μg αCD3ε mAb (BioLegend) per well at 37°C for 5 hours. Human CD4^+^ T cells were then stimulated with plate-bound αCD3ε, soluble αCD28 (5 μg/mL), plus 50 IU/mL human IL-2 (Miltenyi Biotec), 10 ng/mL human rIL-12 (BioLegend), and 100 ng/mL human rIL-27 (PeproTech) in a final volume of 500 μL of media for 72 hours at 37°C in 5% CO_2_.

### Real-time quantitative PCR.

RNA was extracted from sorted human LAG3^–^CD49b^–^, LAG3^–^CD49b^+^, LAG3^+^CD49b^–^ and LAG3^+^CD49b^+^ CD4^+^ T cells. RNA was reverse transcribed to cDNA and real-time qPCR for *IFNG* and *IL10* was performed on a Bio-Rad CFX 384 real-time PCR system (Bio-Rad, Hercules, CA) using the TaqMan Gene Expression Assay for *IFNG* (Assay ID: Hs00989291_m1; Applied Biosystems) and *IL10* (Assay ID: Hs00961622_m1; Applied Biosystems). Relative quantification was performed using the comparative C_T_ method (75) relative to 18S ribosomal RNA (rRNA) (Assay ID: Hs03003631_g1; Applied Biosystems).

### Data and materials availability.

All data are available in the main text and supplemental materials. The human RNA-Seq data are available in the European Genome-Phenome Archive (EGA) database (https://ega-archive.org/) under accession number EGAS00001004454. The results from differential expression analysis between human Tr1 and Th1 cells are available within Supplemental Table 1. The mouse RNA-Seq data are available in the European Nucleotide Archive (ENA) database (https://ebi.ac.uk/) under accession number PRJEB51986. The results from differential expression analysis between mouse Tr1 and Th1 cells are available within Supplemental Table 3.

### Statistics.

Statistical analysis was performed using GraphPad Prism 6 (GraphPad Software). OMIQ Data science platform was used for flow cytometry analysis, including t-distributed Stochastic Neighbor Embedding (tSNE) dimension reduction, Flow Self organizing maps (FlowSOM) clustering, edgeR statistical analysis, and heat map generation. Analysis of human cellular assays was performed using Wilcoxon matched-pairs signed rank test or a 1-way ANOVA to assess more than 2 groups within an experiment. *P* values less than 0.05 were considered significant.

### Study approval.

Human studies were undertaken at Q-Pharm Pty Ltd (Brisbane, Australia) under the approval of the QIMR Berghofer Human Research Ethics Committee (Brisbane, Queensland, Australia; approval P1479). All studies were registered with the Australian and New Zealand Clinical Trial Registration scheme or with the US NIH ClinicalTrials.gov (Supplemental Table 6). Experimental mouse use was in accordance with the “Australian Code of Practice for the Care and Use of Animals for Scientific Purposes” (Australian National Health and Medical Research Council [NHMRC]) and approved by the QIMR Berghofer Medical Research Institute Animal Ethics Committee (Brisbane, Queensland, Australia; approval P2304).

## Author contributions

CLE, SSN, MJB, PZ, GRH, JSM, R Kumar, and CRE conceptualized the study. CLE, SSN, FLR, DC, MMO, TCMF, PTB, SBC, SSS, YW, LB, MSFS, JAE, JN, FHA, JRL, NW, PM, LTK, RLJ, JSL, R Kumar, and JSM developed the methodology and performed experiments and human studies. GRH, JSM, and CRE acquired the funding for the research. FHA, NW, GRH, JSM, and CRE administered the project. FLR, MMO, FHA, NW, MJB, GRH, JSM, and CRE supervised the research. CLE and CRE wrote the original draft manuscript and CLE, SSN, MJB, GRH, JSM, R Kuns, and CRE reviewed and edited the paper. Order of co–first authorship was based on the fact that CLE started the project and performed initial experiments, SSN extended the computational analysis on data sets, and FLR performed all phenotypic and functional validation of data sets.

## Supplementary Material

Supplemental data

Supplemental table 1

Supplemental table 3

Supplemental table 5

Supplemental table 6

## Version 1 01/03/2023

Electronic publication

## Version 2 01/20/2023

title change
