# IL-10–producing Th1 cells possess a distinct molecular signature in malaria

**DOI:** 10.1172/JCI169299

**Published:** 2023-02-15

**Authors:** Chelsea L. Edwards, Susanna S. Ng, Fabian de Labastida Rivera, Dillon Corvino, Jessica A. Engel, Marcela Montes de Oca, Luzia Bukali, Teija C.M. Frame, Patrick T. Bunn, Shashi Bhushan Chauhan, Siddharth Sankar Singh, Yulin Wang, Jinrui Na, Fiona H. Amante, Jessica R. Loughland, Megan S.F. Soon, Nicola Waddell, Pamela Mukhopadhay, Lambros T. Koufariotis, Rebecca L. Johnston, Jason S. Lee, Rachel Kuns, Ping Zhang, Michelle J. Boyle, Geoffrey R. Hill, James S. McCarthy, Rajiv Kumar, Christian R. Engwerda

Original citation: *J Clin Invest*. 2023;133(1):e153733. https://doi.org/10.1172/JCI153733

Citation for this corrigendum: *J Clin Invest*. 2023;133(4):e169299. https://doi.org/10.1172/JCI169299

The title of this paper was incorrect. The correct title is above. The HTML and PDF versions have been updated online. 

The authors regret the error. 

## Version 1 02/15/2023

Electronic publication

