# Supplementary material for: IL-10-producing Th1 cells possess a distinct molecular signature in malaria
Source: J Clin Invest. 2023 Jan 3;133(1):e153733. doi: 10.1172/JCI153733 (PMC9797345; doi:10.1172/JCI153733)
Supplement: Supplemental data [file jci-133-153733-s008.pdf]

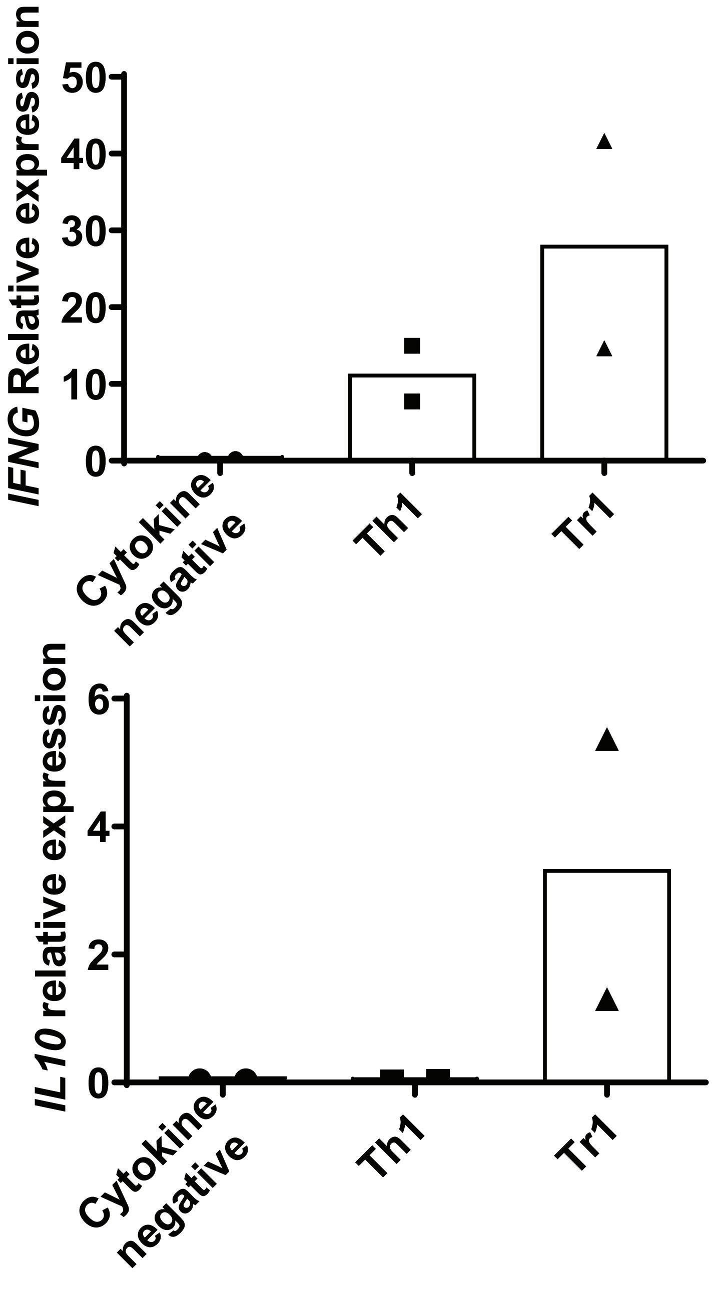

Figure S1. Related to Figure 1. *IFNG* and *IL10* mRNA expresion in sorted controlled human malaria infection cell populations. Cytokine-negative, Th1 and Tr1 cells were isolated as outlined in Figure 1. Excess RNA isolated from n = 2 individuals samples at day 14 post-infection were assessed for *IFNG* and *IL10* mRNA expression.

A

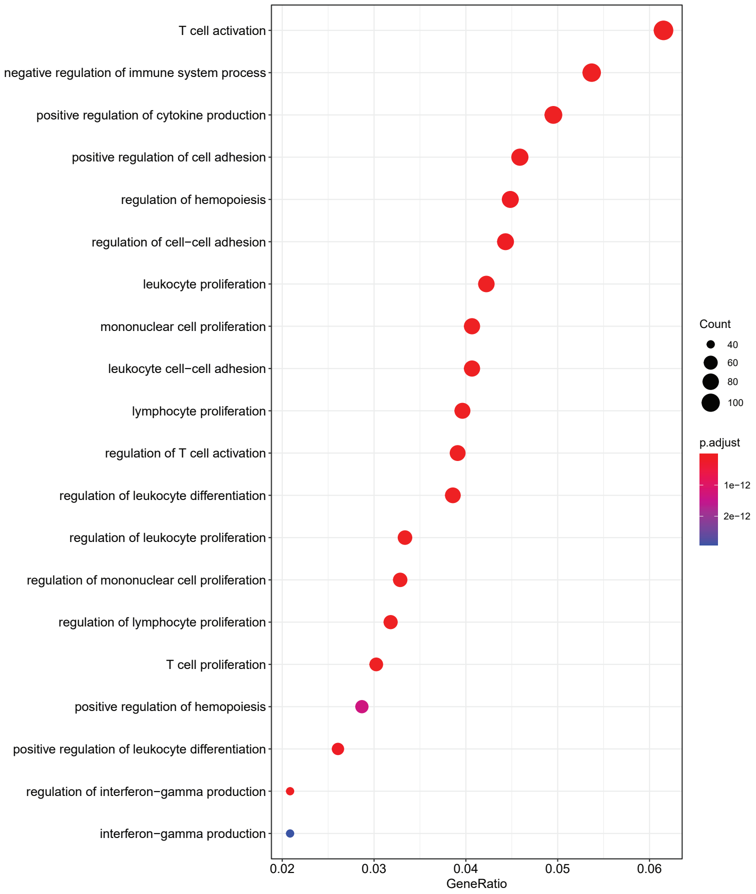

B

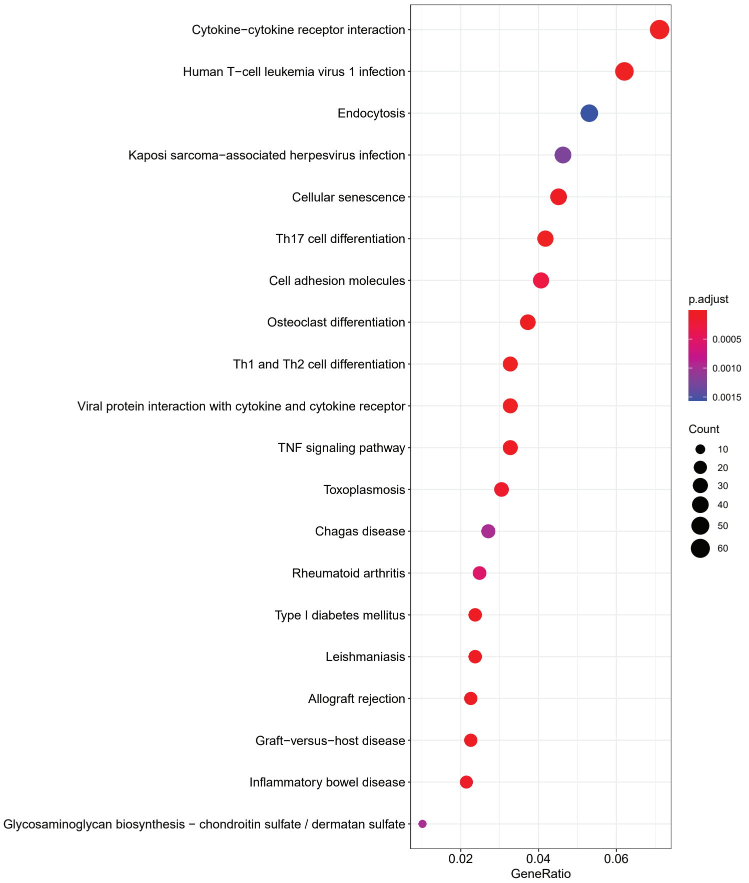

Figure S2. Related to Figure 3. Enriched categories from Gene Ontology analysis and KEGG pathway analysis of the mouse Tr1 cell gene signature. The top 20 enriched GO terms from sub-ontology Biological Process (A) and top 20 enriched KEGG pathways (B) based on comparison of mouse Tr1 and Th1 cells. Differentially expressed genes identified from Tr1 versus Th1 cells (FDR < 0.05) were used as inputs to each analysis.

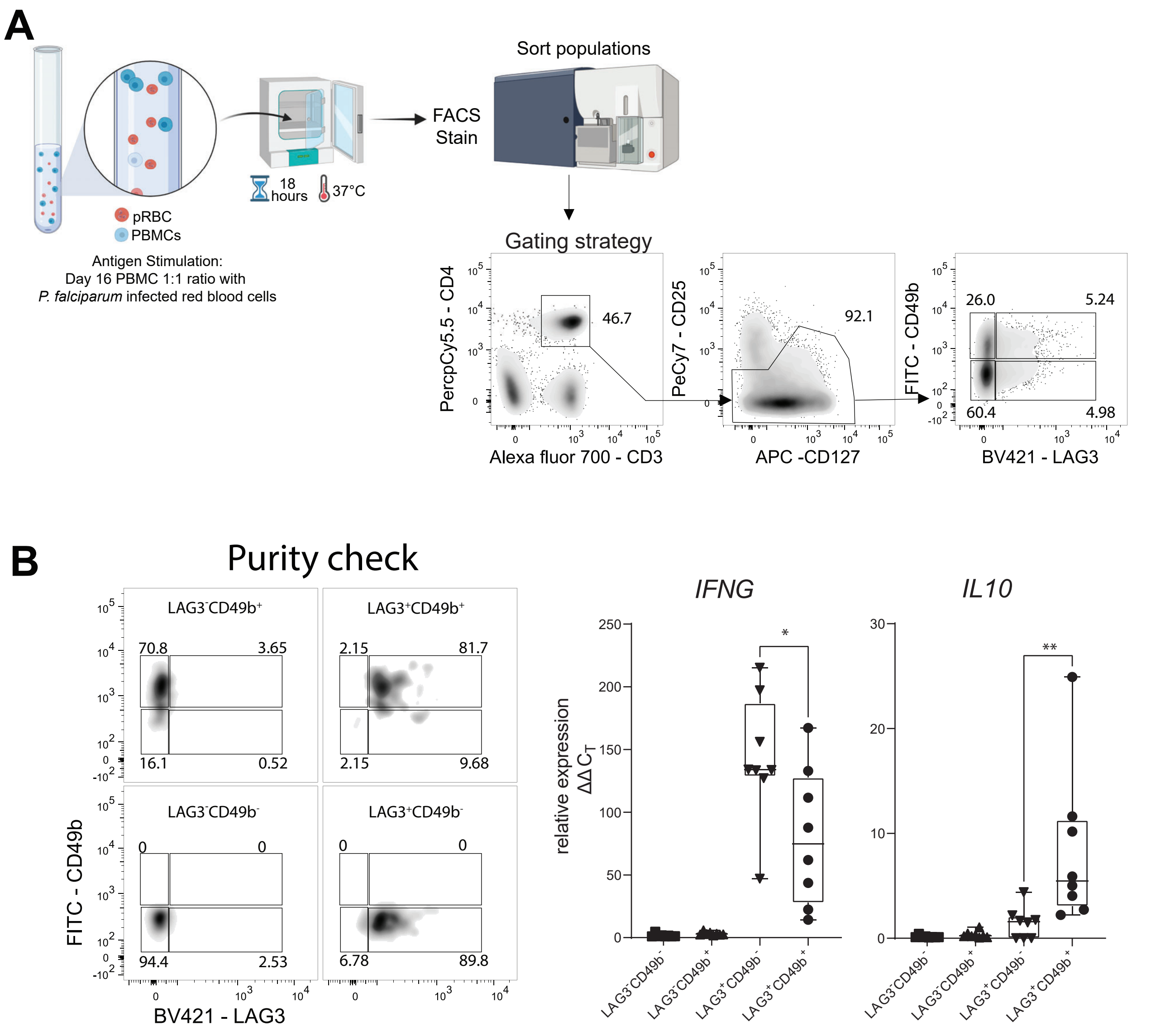

Figure S3. Related to Figure 5. LAG3$^+$ CD49b$^+$ CD4$^+$ T cells represent Tr1 cells in controlled human malaria infection (CHMI). Peripheral blood mononuclear cells from CHMI volunteers taken on day 16 post-infection were stimulated with parasitised red blood cells for 18 hours, prior to sorting non-regulatory T cells (CD25^{lo}), based on LAG3 and CD49b expression (A). RNA was isolated from purified CD4$^+$ T cell subsets and *IFNG* and *IL10* mRNA expression was measured (B). Cells isolated from n = 8 volunteers, *P < 0.05, ** P < 0.01. Significance assessed by Wilcoxon matched-pairs signed-rank test.

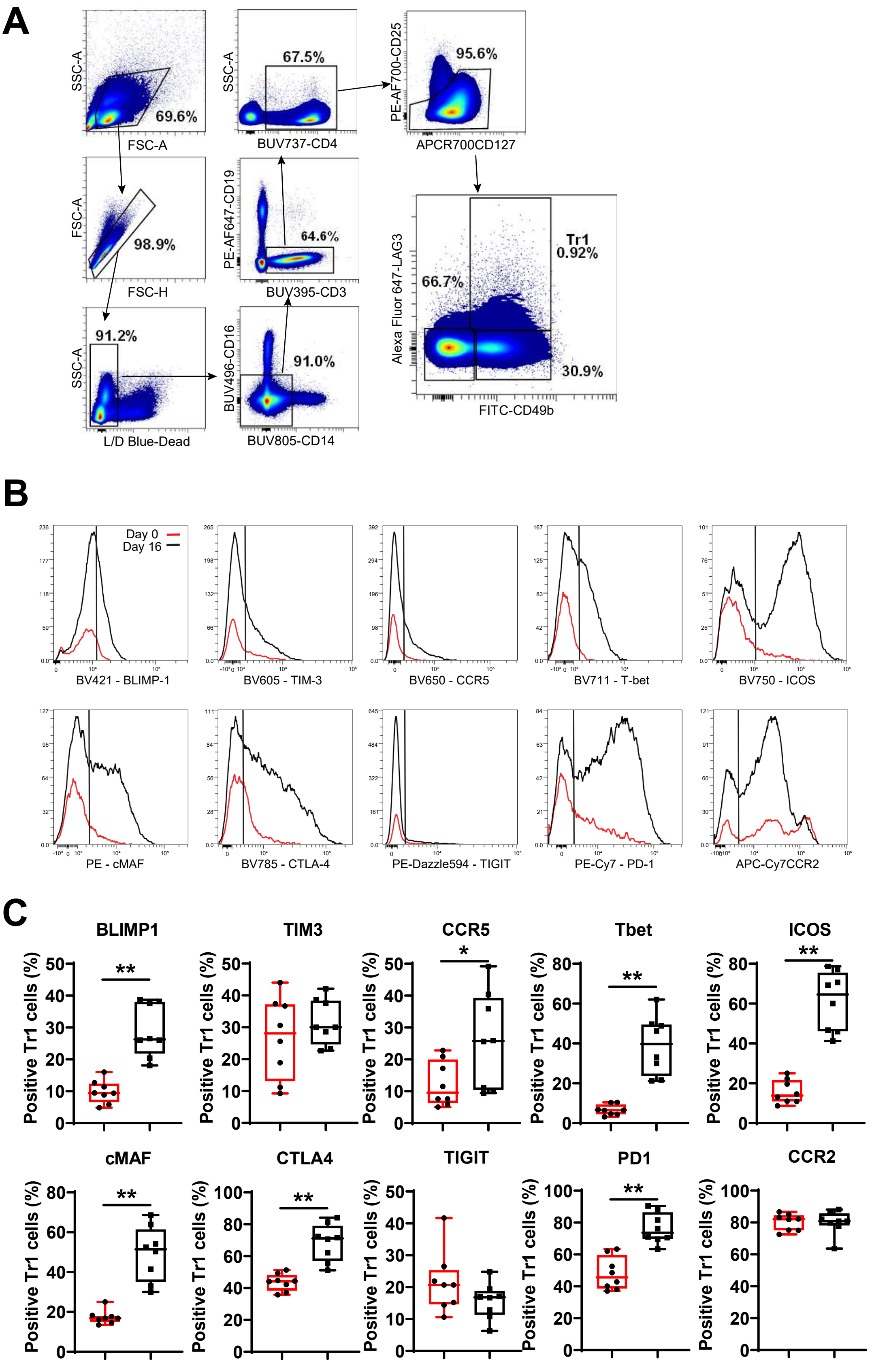

Figure S4. Related to Figures 5 and 7. Gating strategy used to measure the expression of molecules associated with human Tr1 cells from humans infected with *Plasmodium falciparum* (A). The expression of co-inhibitory and chemokine receptors was assessed on gated Tr1 cells at day 0 (red lines) and day 16 (black lines) post-infection, with data shown as concatinated histograms (n = 8 paired samples) (B). The percentage positive Tr1 cells for each marker at day 0 (red box) and day 16 (black box) post-infection are shown as box and whisker plots indicating median + minimum and maximum (n = 8 paired samples) (C), *P < 0.05 and **P < 0.01. Significance assessed by Wilcoxon matched-pairs signed rank test.

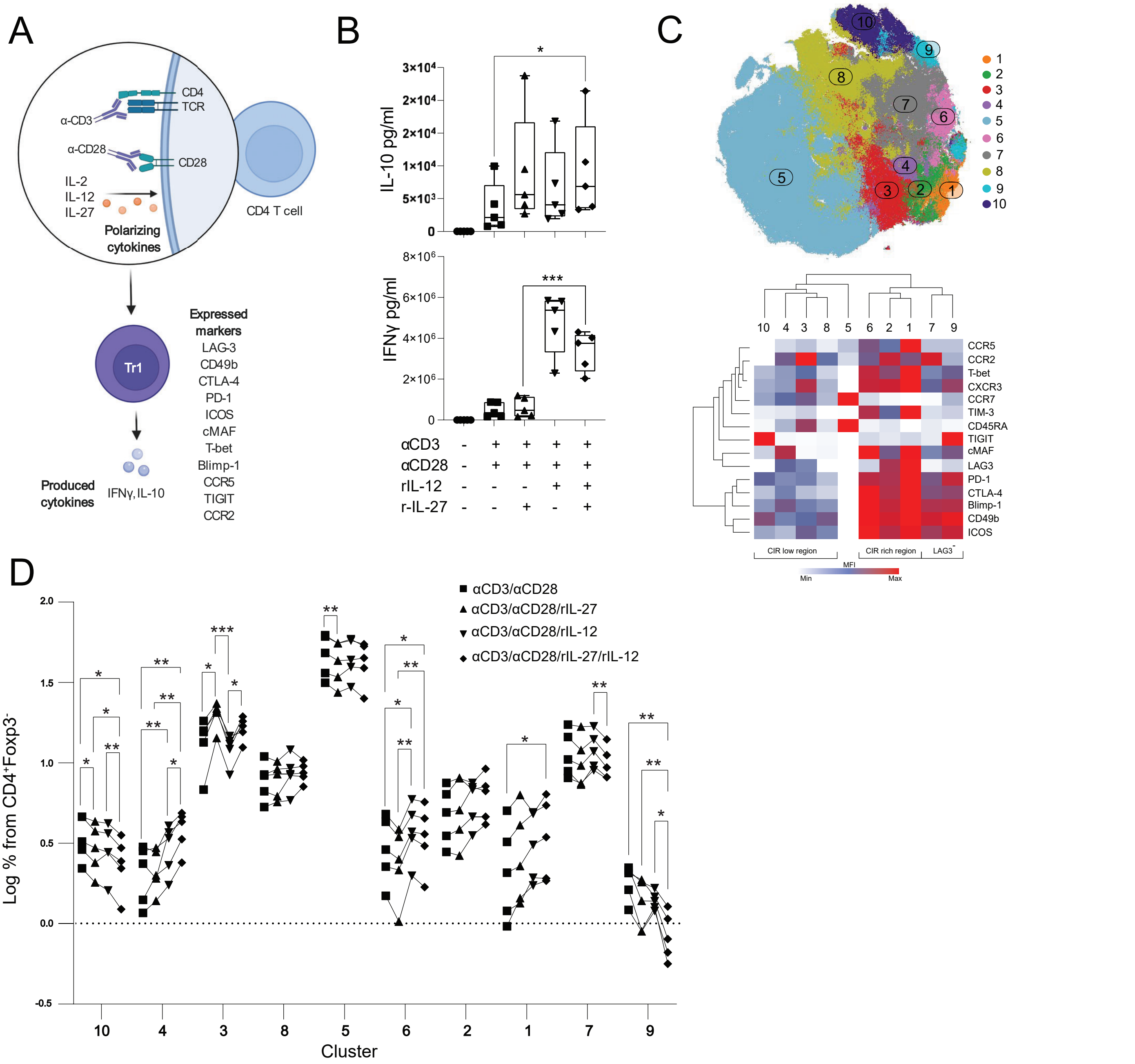

Figure S5. Related to Figure 7. Activation of primary human CD4+ T cells in vitro. Primary human CD4+ T cells stimulated with anti-CD3ε and anti-CD28 monoclonal antibodies were polarised to a Tr1 cell phenotype as outlined (A) with different combinations of recombinant IL-2, IL-12 and IL-27 for 72 hours before measuring IL-10 and IFNγ levels in cell culture supernatants (B). Clustering of FoxP3-negative CD4+ T cells based on expression of Tr1 cell-associated molecules by tSNE plot was performed and the mean fluorescence intensity of staining for all 10 cell clusters identified was presented as a heat map (C), along with the relative frequency of each cluster amongst FoxP3-negative CD4+ T cells (D). n = 5 paired volunteer samples in each treatment group; *$p<0.05$, **$p<0.01$ and ***$p< 0.001$ assessed by paired one-way ANOVA (B and D).

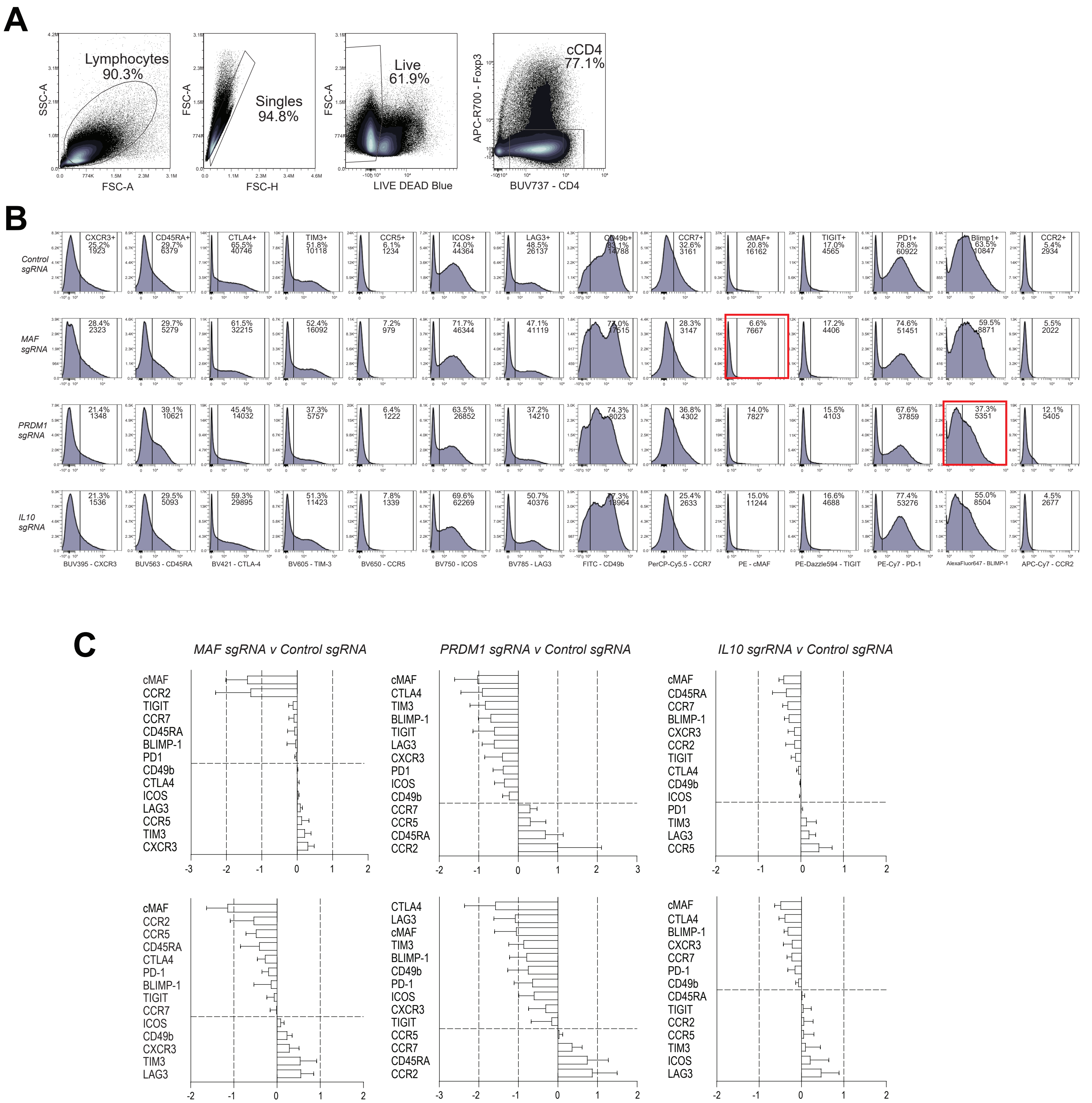

Figure S6. Related to Figure 7. The effects of *MAF*, *PRDM1* and *IL10* deletion on co-inhibitory receptor expression. Following gene editing, as indicated, and 72 hours of culture with anti-CD3ε and anti-CD28 monoclonal antibodies, plus IL-2, IL-12 and IL-27, FOXP3^{-} CD4^{+} T cells were identified by flow cytometry (A). The expression of co-inhibitory and chemokine receptors were assessed, numbers in concatinated (n = 4) histograms indicate percentage positive cells and mean fluorescent intensity (MFI). Red boxes indicate cMAF and BLIMP-1 expression in corresponding gene-engineered CD4^{+} T cells (B). Changes in the percentage positive (top) and expression (MFI; bottom), shown as log-fold change; n = 4 paired samples per treatment group, mean +/- SE (C).
