# Supplementary material for: IL-10-producing Th1 cells possess a distinct molecular signature in malaria
Source: J Clin Invest. 2023 Jan 3;133(1):e153733. doi: 10.1172/JCI153733 (PMC9797345; doi:10.1172/JCI153733)
Supplement: Supplemental table 5 [file jci-133-153733-s011.pdf]

Table S5. Human malaria trial cohort demographics

Trial registration code	*Year of study*	*Trial cohort*	*Age (Years)*	*Gender*
ACTRN12613000565741	2014	3A	24	M
			20	F
			22	M
			25	F
			19	F
		3B	18	M
			22	F
			32	F
			23	M
NCT02281344	2014	1	29	M
			24	M
			19	M
NCT02389348	2015	1	19	F
			24	F
NCT02431650	2016	1	55	M
			26	M
			29	M
		2B	26	M
			20	M
		3	34	M
			26	M
			20	M
NCT02431637	2016	1	20	M
			26	M
		3	32	F
			22	F
NCT02573857	2015	1	32	M
NCT02867059	2016	1	45	M
			27	M
			21	M
			21	M
			22	M
			23	M
			30	M
NCT02783833	2016	B1	24	M
			29	M
			24	M
			34	M
			47	M
			23	M
			34	M
		B2	23	M
			31	M
			48	M
			21	M
			38	M
			39	M
			27	M
			20	M
NCT03542149	2018	1	18	M

			29	M
			23	M
			22	F
			23	M
			22	M
			22	F
			21	F
